# Exogenous RNAi mechanisms contribute to transcriptome adaptation by phased siRNA clusters in *Paramecium*

**DOI:** 10.1093/nar/gkz553

**Published:** 2019-06-28

**Authors:** Sivarajan Karunanithi, Vidya Oruganti, Simone Marker, Angela M Rodriguez-Viana, Franziska Drews, Marcello Pirritano, Karl Nordström, Martin Simon, Marcel H Schulz

**Affiliations:** 1 Cluster of Excellence, Multimodal Computing and Interaction, Saarland University and Department for Computational Biology and Applied Algorithmics, Max Planck Institute for Informatics, Saarland Informatics Campus, 66123 Saarbrücken, Germany; 2 Graduate School of Computer Science, Saarland Informatics Campus, 66123 Saarbrücken, Germany; 3 Institute for Cardiovascular Regeneration, Goethe-University Hospital, 60590 Frankfurt, Germany; 4 Molecular Cell Dynamics, Centre for Human and Molecular Biology, Saarland University, 66123 Saarbrücken, Germany; 5 Molecular Cell Biology and Microbiology, Wuppertal University, 42097 Wuppertal, Germany; 6 Genetics/Epigenetics, Centre for Human and Molecular Biology, Saarland University, 66123 Saarbrücken, Germany

## Abstract

Extensive research has characterized distinct exogenous RNAi pathways interfering in gene expression during vegetative growth of the unicellular model ciliate *Paramecium*. However, role of RNAi in endogenous transcriptome regulation, and environmental adaptation is unknown. Here, we describe the first genome-wide profiling of endogenous sRNAs in context of different transcriptomic states (serotypes). We developed a pipeline to identify, and characterize 2602 siRNA producing clusters (SRCs). Our data show no evidence that SRCs produce miRNAs, and in contrast to other species, no preference for strand specificity of siRNAs. Interestingly, most SRCs overlap coding genes and a separate group show siRNA phasing along the entire open reading frame, suggesting that the mRNA transcript serves as a source for siRNAs. Integrative analysis of siRNA abundance and gene expression levels revealed surprisingly that mRNA and siRNA show negative as well as positive associations. Two RNA-dependent RNA Polymerase mutants, RDR1 and RDR2, show a drastic loss of siRNAs especially in phased SRCs accompanied with increased mRNA levels. Importantly, most SRCs depend on both RDRs, reminiscent to primary siRNAs in the RNAi against exogenous RNA, indicating mechanistic overlaps between exogenous and endogenous RNAi contributing to flexible transcriptome adaptation.

## INTRODUCTION

RNA interference (RNAi) is a conserved eukaryotic mechanism, which involves small non coding RNAs to regulate gene expression and genome integrity. Among the broad variety of small RNA biogenesis pathways and functions, micro RNAs (miRNAs) and short interfering RNAs (siRNAs) are two of the best studied classes (1). The latter are usually produced from double-stranded RNA (dsRNA) precursors arising from bidirectional transcription or activity of a RNA-dependent RNA polymerase (RDR). This dsRNA then acts as a substrate for Dicer, a RNAse III domain containing enzyme, which cleaves dsRNA into discrete siRNA duplexes. When the siRNA duplexes are cleaved in a regular interval from one precursor, the process is called phasing, which was first discovered in plants (2).

In contrast, miRNAs are derived from hairpin RNAs, thus being independent of bidirectional transcription or RDRs. The stem of the hairpin is cut twice by Dicer in plants or by Drosha (a special RNAse III enzyme) and Dicer in mammals, generating a miRNA duplex (3). As the miRNA pathway is independent of RDRs, a single stranded RNA folds back to create a RNAse III substrate, many animals were believed to have lost their RDRs with exception of the nematode model *Caenorhabditis elegans*. However recent findings suggest the existence of RDRs in many other animals, where they could contribute to RNAi in yet unknown mechanisms (4). Parallel to the loss of canonical RDRs, mammals were previously believed to have lost their virus-targeting RNAi, but evolved protein based antiviral mechanism by interferon response. This hypothesis is now under debate as recent papers showed RNAi-dependent small RNAs against viral infections (5,6). Even more intriguing, the interferon negative mammalian cells show efficient dsRNA-induced silencing, which is abolished by treatment with type I interferon (7). Consequently, we need to re-evaluate the stereotyped thinking about evolutionary loss of RDRs and antiviral RNAi, which highlights the importance of studying underlying mechanisms. RDRs are thus in the spotlight as their functions are highly diverse in different organisms (4). RDRs have mainly been described to be involved in amplification of RNAi. In plants, long single stranded RNAs (mRNA or virus RNA) become converted into long dsRNA Dcr substrates by RDRs: this can be initiated by miRNA/siRNA attack or by unknown mechanisms in case of viral RNA (8). These phased secondary (2^*o*^) siRNAs have been shown to act in trans, thus controlling complex regulatory networks (9). In *C. elegans*, 2^*o*^ siRNAs are produced by RDRs in a Dicer-independent manner as direct RDR transcripts from mRNA: this was found to be conserved as a response to exogenous dsRNA, viral RNA and also endogenous triggers (10–14). In comparison, both plants and nematodes, use RDRs for exo- and endogenously triggered 2^*o*^ siRNA amplification, attempting also to produce enough siRNAs for systemic distribution.

In *Paramecium*, in contrast to plants and nematodes, primary (1^*o*^) siRNAs depend on RDR activity. It was shown that two RDRs, RDR1 and RDR2, are necessary to produce 1^*o*^ siRNAs from exogenously introduced dsRNAs (15,16). This dependency was shown by recursive RNAi, and distinct mutant analysis of the two RDRs, RDR1 and RDR2. A surprising fact here is that the exogenous dsRNA becomes amplified first instead of directly getting diced into siRNAs. In addtion, 2^*o*^ siRNAs were less abundant than 1^*o*^ siRNAs, which may be related to the fact that there is no need to systemically distribute siRNAs among tissues. In these unicellular organisms, it is unknown to which extent components of the exogenous pathways also control endogenous siRNAs and as a result transcriptome dynamics.

For this reason we describe here the small RNA world of vegetative *Paramecium*, a longstanding model system for epigenetic phenomena (17). Taking advantage of the nuclear dimorphism, representing germline micronuclei and the somatic macronucleus in a single cell, these cells phenotypically show several instances of epigenetic inheritance of gene expression, such as mating type or serotype determination and inheritance (18). Small RNAs have been extensively studied during development, which unraveled the involvement of scnRNAs and iesRNAs in the programmed excision of transposon derived sequences during development of the new macronucleus after sexual recombination (19–22). During vegetative growth, it has been demonstrated that distinct RNAi pathways occur simultaneously in growing cultures: one using exogenous dsRNA from food bacteria that attacks mRNAs on the post-transcriptional level (15,23) and another pathway in which truncated transgenes can silence homologous endogenous remote loci at the chromatin level (15,24). Both pathways, dsRNA feeding and transgene induced, accumulate siRNAs of a predominant length of 23 nt and involve activity of RDRs. Analysis of small RNAs during vegetative growth was until now not carried out on a genome-wide level, but restricted to the analysis of exogenously triggered RNAi against endogenous genes.

In this study, we analyzed and characterized genome-wide small RNAs of *Paramecium tetraurelia* in context of their global dynamics in different serotypes. We analyzed the small RNA diversity of *Paramecium* by deep sequencing of various RNA samples from different environmental conditions. Our analysis indicates that *Paramecium* does not possess canonical miRNAs, but instead we can identify many genes regulated by phased siRNAs produced from the entire gene. Our subsequent analyses of two different RDR mutants indicate that these clusters depend on both RDRs, which is the same for 1^*o*^ siRNAs produced from exogenous siRNAs. The comparison of their function in endogenous and exogenous RNAi let us conclude that they synergistically provide dsRNA Dicer substrates from single stranded and double stranded endo- and exogenous RNA. We conclude, that in contrast to other organisms, the role of RDRs in *Paramecium* focuses more on the recognition and dissection of RNA substrates and thus on the initiation of RNAi rather than providing an amplified signal in the form of massive amounts of secondary siRNAs.

## MATERIALS AND METHODS

### Cell culture, RNAi by feeding

*Paramecium* cells of stock 51 and stock d42 were cultured as described before using *Klebsiella pneumoniae* for regular food in WGP (Wheat grass powder, Pines International Co., Lawrence, KS, USA) medium. Serotype pure cultures were maintained at 14°C (51H), 24°C (51B, 51D) and 31°C (51A). Serotype expression was verified by immobilization with homologous polyclonal antisera (rabbit anti-51A/-51B/-51D/-51H). Developmental stages of cells were analysis by DAPI staining of nuclei to verify the vegetative stage of cultures. RNAi by feeding of dsRNA producing bacteria was carried out as described before using the double T7 vector L4440 in the RNAse III deficient *Escherichia coli* HT115DE3 (25).

### RNA isolation, library preparation and sequencing

Total RNA was isolated with TriReagent (Sigma-Aldrich, Seelze, Germany) and integrity was checked by denaturing gel electrophoresis after DNAse I (Invitrogen, Karlsruhe, Germany) digestion and subsequent purification with acid phenol. For siRNA sequencing, 17–25 nt small RNA fractions were isolated by denaturing polyacrylamide gel electrophoresis and subjected to standard small RNA libraries using the NEB Next small RNA sequencing Kit (NEB, Frankfurt a.M., Germany). The procedure includes 3′-OH and 5′-monophosphate specific ligation steps and we tried to lower 3′-2′-O-me biases by 18 hours 3′-ligation at 16°C. After 10 polymerase chain reaction cycles, the libraries were gel-purified and sequenced on the HiSeq 2500 using the Rapid Mode with 28 cycles. Reads were de-multiplexed and adapter sequences were trimmed using Trim Galore (http://www.bioinformatics.babraham.ac.uk/projects/trimgalore/ ) that uses Cutadapt (26) with a stringency cutoff of 10.

### RDR mutant strains

We analyzed two mutant strains, Rdr1 and Rdr2, originating from a forward genetic screen for cells with defective RNAi induced by application of exogenous dsRNA (16). We used the rdr1-5.28 line with a putative null allele for Rdr1, a frameshift mutation leading to a premature stop codon before the catalytic domain. For Rdr2, we used the rdr2-5.32 line harboring a missense mutation CGA to a TGA stop codon inside the catalytic domain (Supplementary Figure S1).

### Dataset description and retrieval

We categorize the datasets in our study into two groups: (i) Cluster definition data, and (ii) Analysis data.

#### Cluster definition data

We sequenced sRNA-seq datasets, with two replicates for each wild-type (WT) serotype 51A, 51B, 51D and 51H to characterize small RNA clusters (SRCs). However, we did not have paired mRNA-seq data from the respective biological sample.

#### Analysis data

Expression of the *P. tetraurelia* (Stock 51; version 2) mRNA transcripts for the four WT serotypes (51A, 51B, 51D, 51H; three replicates each) was obtained from our recent study (27) (European Nucleotide Archive (ENA) Accession: PRJEB9464). In order to have a consistent downstream analysis, we sequenced small RNAs (four WT serotypes, three replicates each) from the same biological replicates to obtain sRNA expression data paired with the existing mRNA data. For RdRP mutants, we sequenced three replicates (51A-Rdr1, 51A-Rdr2) of sRNA, and mRNA data. The datasets created as part of this study can be accessed at ENA (Accession: PRJEB25903).

### Read alignment and cluster generation

Small RNA reads of length 21–25 nt were aligned using the alignment module from ShortStack (version 3.4) (28), with default parameters. Reads shorter than 21 nt were not considered as they are potential RNA degradation products. Reads were aligned against the *P. tetraurelia* MAC genome (version 2;stock 51). In order to control for sequencing depth each aligned dataset was downsampled, such that they had an equal number of alignments. The cluster calling module of ShortStack was used to identify novel SRCs from the downsampled alignments using a minimum alignment coverage parameter (*mincov*) set to 20 alignments (Effect of the coverage parameter can be seen in Supplementary Table S1). The padding parameter (*pad*) was set to 100 bp, such that distinct clusters within 100 bp are merged into one cluster. We note that ShortStack3 uses a probabilistic algorithm to place multi-mapping sRNA reads, which was used with the default values (bowtie_m=50, mmap=u) to improve the amount of used reads (28).

All identified clusters from each serotype were unified using mergeBed (from BEDtools v2.23; default parameters) (29) into one consistent annotation set, denoted SRCs, which allowed unbiased comparison across serotypes.

Quantification of sRNA accumulation in the identified SRCs was done using the RAPID software (30) (https://github.com/SchulzLab/RAPID). RAPID was run with default parameters, which only considers error-free alignments but allows multi-mapping reads (-k 100; -k is the bowtie2 parameter controlling the number of multi-mapping reads to be reported in alignments).

### Normalization of sRNA reads

For all comparative analyses we used the normalized counts, and converted these values to Transcripts Per Million (TPM), which we also refer to as sRNA accumulation. For the analyses specific to individual WT serotypes, SRCs with a TPM value greater than one were termed as *serotype specific SRCs*. We used RAPID to obtain the normalized counts, which implements the KnockDown Corrected Scaling (KDCS) method (30), which was previously developed to normalize small RNA read counts and adjust for feeding associated small RNAs (24).

### Differential expression analysis

We performed a differential expression analysis of the SRCs between WT serotype (51A), and each mutant (51A-Rdr1, 51A-Rdr2) separately. The raw small RNA read counts of SRCs are subjected to differential analysis, using the R/Bioconductor package DESeq2 (version 1.18.1) (31). Following the DESeq2 analysis, SRCs with a false discovery rate lesser than 0.05 (*FDR* < 0.05) are considered to be SRCs with statistically significant differential expression.

### Boundary modification of SRCs

Investigation of SRCs in the IGV Browser (version 2.3.91) (32) showed occurrences of non-specific boundaries. For instance, one SRC region could overlap with more than one gene (Supplementary Figure S2; see detailed description in Supplementary Methods). Hence, before comparing small RNA accumulation with the gene expression data, boundaries of such non-specific SRCs needed to be modified, to eliminate non-specific pairs of SRC-gene overlaps from further analysis. All SRCs were overlapped against the MAC genome annotation of *P. tetraurelia* (Stock 51; version 2). SRCs which did not overlap with any gene were removed for this analysis.

In a gene-SRC overlap, if the gene was covered by more than 80% and the SRC was covered at least by 20%, then the SRC’s boundary was limited to the gene’s boundary. This removed non-specific gene overlaps. However, this condition also removed genes which can overlap with multiple SRCs. In order to account for such cases, another condition was introduced. If the gene was covered at least by 10% and the SRC was covered by more than 80%, such SRCs are retained without any boundary changes. The sRNA accumulation of these boundary modified SRCs were requantified and normalized using RAPID.

### Comparison of small RNA with mRNA data

In order to correlate sRNA accumulation with mRNA expression in genes, we needed to handle two special cases in the overlap of SRCs and genes. First, when a gene mapped to multiple SRCs we summed the sRNA accumulation of all the SRCs mapping against that gene. Second, when a SRC mapped to multiple genes, we simply associated the sRNA accumulation of that SRC against each gene it maps to. After mapping SRCs to genes, for each gene, we calculated the correlation between mRNA expression (TPM), and sRNA accumulation (TPM) by utilizing all WT serotype (51A, 51B, 51D, 51H) replicate measurements. However, for serotype specific analysis, we used an aggregate sum of respective replicates. Expression of mRNA was quantified using Salmon (version 0.8.2) (33).

#### Quantification of sRNA in exon–exon junctions and introns

To investigate the source of sRNA, we quantified the accumulation of sRNA in the exon–exon junctions (EEJ), and introns. We obtained the list of introns from the ParameciumDB (34). Using the exon information from the MAC genome annotation (version 2; stock 51), we defined an EEJ as 18 bps upstream and downstream of an exon–exon boundary.

### Phasing prediction

For the prediction of phased regions from small RNA read alignments we have used the established phase score (*P*-score) method (35). Here, the *P*-score is used to compute the enrichment of aligned siRNA reads on both strands in phased registers using a window of size 253 bps, considering 11 registers of length 23, the predominant siRNA length in *Paramecium*. Each register contains 23 bins, one phased bin and 22 non-phased bins. All windows with a *P*-score > 10 are predicted to be phased. In addition, there must be RNA reads in at least 3 distinct phased bins (out of 22 possible) for each strand and a minimum total of 20 reads per window. Phased regions that are within 100 bps were combined into one region. For the phasing prediction the down-sampled read libraries were used in order to compare predictions between serotype samples.

### Annotation of SRCs

We utilized the genome annotation file of *P. tetraurelia* (Stock 51; version 2) downloaded from the parameciumDB (paramecium.cgm.cnrs-gif.fr) to annotate the identified *serotype specific SRCs*. We used BEDtools (intersectBed; version 2.23) to identify the annotation categories, and custom R scripts to plot the annotation results. Annotations mapping to the number of protein-coding genes were handled separately, after performing SRC boundary modification.

#### rRNA annotation

For ribosomal RNA analysis we used the rRNA cluster published by (36): GenBank Accession: AF149979.1, with the additional annotation of the 5.8S sequence by (37): GenBank accession: AM072801.1.

#### Pseudogene annotation

As pseudogenes were not part of the annotation, we used the pseudopipe (38) software to predict pseudogenes. We utilized the default parameters except we adapted for the specific genetic code in *Paramecium* for the tblastn step of the software.

### Gene ontology enrichment

We used the Gene Ontology (GO) association file downloaded from the paramecium DB (http://paramecium.cgm.cnrs-gif.fr/download/species/ptetraurelia/v2/functional/) and performed GO enrichment analysis using Ontologizer (version 2.0) software (39). We used the parent-child union method, and Benjamini–Hochberg correction for analyses. We considered GO terms with a multiple testing corrected *P*-value < 0.05 as statistically significant.

## RESULTS

### Definition of endogenous small RNA clusters (SRCs) reveals siRNAs but no miRNAs

To characterize the small RNAs of *Paramecium* and their dynamics during environmental alterations, we isolated RNA of vegetatively growing paramecia at different temperatures and serotypes. *Paramecium* undergoes antigenic variation similar to pathogenic protists; exclusive expression of surface antigen genes are associated with environmental parameters such as cultivation temperature (17,40) and we have previously shown that individual serotypes are associated with massive transcriptome alterations, rather than switching of the surface antigen only (27). We therefore used four serotype pure cultures (51A, 51B, 51D, 51H), which result from long term cultivation at 31°C for 51A, 26°C for 51B and 51D, and 14°C for 51H. For small RNA analysis, 17-25 nt fractions were gel purified and subjected to library preparation.

**Figure 1.**
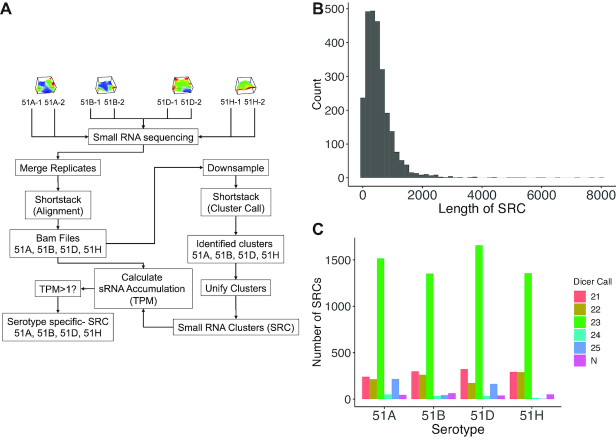
(**A**) Overview of the small RNA cluster (SRC) generation workflow. The first row visualises the different serotypes according to a transcriptome analysis done in (27). (**B**) Length distribution of SRCs. (**C**) Number of serotype specific SRCs (*y*-axis) detected in the WT serotype samples (replicates were merged), stratified according to the predominant small RNA length (dicer call), where N means that no predominant length could be found.

As it has not been investigated yet how prevalent small RNA expression is in the *Paramecium* genome during vegetative states, we created a bioinformatics workflow to detect SRCs, as shown in Figure 1A. We sequenced two biological replicates for each serotype described above. After merging both replicate datasets, we aligned the reads to the macronucleus genome (*P. tetraurelia*, stock 51; version 2) using ShortStack (28). In order to make predictions comparable between different sequencing runs, we downsampled all alignments for a serotype to be of equal size and identified SRCs for each serotype. ShortStack predicts miRNA precursors *de novo* using RNA folding, and searching for stem loops, but no clusters were predicted as miRNAs in our data. To ease comparison across different serotypes, we unified all clusters obtained from each serotype to a set of SRC. For these SRCs, we quantified small RNA accumulation in each serotype and identified *serotype-specific SRCs*, which had an accumulation of at least one TPM in the respective serotype.

### The 23 nt SRCs follow transcriptome dynamics

Using this strategy, we identified 2602 SRCs after unifying data from all serotype samples (Supplementary Table S1). Figure 1B shows the length distribution of all SRCs. The majority showed a genomic expansion between 100 and 1000 bps, with a smaller set of clusters larger than 2000 bps. The predominant small RNA length observed for the majority of clusters is 23 nt, which is in agreement with previous reports of exogenously triggered RNAi pathways in *Paramecium* (Figure 1C). The number of expressed SRCs was similar in all serotype samples (51A: 2286, 51B: 2058, 51D: 2393, 51H: 2012). We used a clustering analysis to compare sRNA accumulation of all SRCs between serotypes. The clustered heat map in Supplementary Figure S5 shows, that individual serotypes can be distinguished according to the abundance of small RNAs in the defined SRCs. Hence, we are able to differentiate different transcriptomic states according to their SRC expression.

### The majority of SRCs are in protein-coding genes

**Figure 2.**
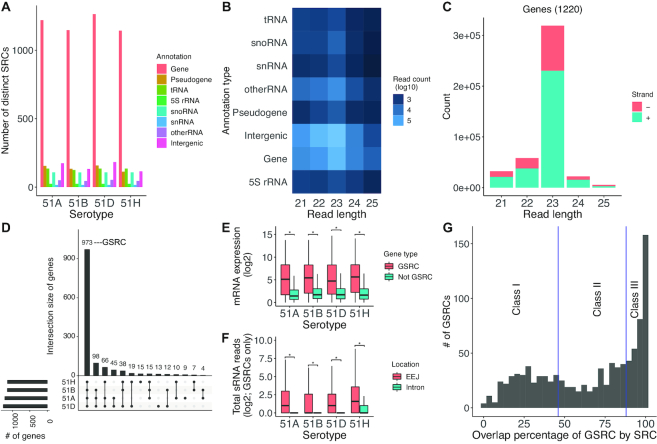
(**A**) Serotype specific SRCs expressed in the WT serotype (replicates were merged) samples were overlapped with annotated regions. Each annotated element is counted only once (distinct counting) and the number of elements of the different types is shown on the *y*-axis for all four serotypes. (**B**) sRNA reads (log10) in SRCs overlapping different genomic annotations (rows) and restricted to small RNA length (*x*-axis) for 51A serotype. (**C**) Length distribution of sense and antisense sRNAs mapping to protein-coding genes in 51A serotype. (**D**) Set intersection plots for SRCs overlapping with genes across serotypes. Genes consistently overlapping in the four WT serotypes, are called Genes associated with SRCs (GSRCs). (**E**) Boxplot of mRNA expression (*y*-axis, log2 TPM) in the four WT serotypes of GSRCs and other expressed genes. (**F**) Boxplot of total sRNA reads (*y*-axis; log2) in the EEJ and introns of GSRCs. (**G**) A histogram of the number of GSRCs (*y*-axis) plotted against the overlap percentage (in basepairs) of a GSRC by one or more SRCs (*x*-axis) is shown here. Based on the overlap percentage, GSRCs were split into three classes as shown (vertical lines) for further analysis. *P < 0.05 with a two-tailed Wilcoxon test.

To clarify which genomic regions produce small RNAs, SRCs were overlapped with annotated regions of the *Paramecium* genome (41,42). Taking advantage of the recent correction of gene annotation (43), regions were classified for genes (ORFs), intergenic regions, tRNAs, 5SrRNA, snoRNAs and snRNAs. In this context, the rDNA cluster producing the 17S, 5.8S and 25S ribosomal RNAs was analyzed separately being not part of the genome annotation but producing a considerable amount of small RNAs (see below). Figure 2A shows the number of SRCs in individual serotype samples, that overlap distinct annotated regions. Importantly, it was found that the majority of ∼1300 SRCs associate with regions of protein-coding genes, suggesting a potential involvement with gene expression regulation as discussed below.

At first glance, only few clusters can be found in non-coding RNA loci, however, also fewer loci are annotated as non-coding. For example, in Serotype 51A, 1220 SRCs were in genes (∼3% of 40 460), 117 in pseudogenes (∼5% of 2435 pseudogenes), 212 in intergenic regions (∼0.4% of 39 156), 135 in tRNAs (∼68% of 198 annotated tRNAs), 16 in snRNAs (100%), 24 in 5S-rRNAs (∼96% of 25 5S rRNAs), 108 in snoRNAs (∼76% of 142 annotated snoRNAs) and 50 in the category of other RNAs with diverse functions (∼7.2% of 689 other RNA loci). We can rule out the possibility that we included mature tRNAs, snoRNAs and 5S RNAs in our library because we size-selected to input RNA below 25 nt. Furthermore, the preference for 23 nt siRNAs of these SRCs together with the occurrence of antisense reads let us conclude that these siRNAs are specifically produced by the RNAi machinery.

We investigated the read length and strand distribution of reads for all annotation types. All these SRCs have a predominant read length of 23 nt. (Figure 2C; data shown for Serotype 51A, detailed data for all serotypes in Supplementary Figure S6). As the number of identified clusters does not allow for a general quantification of siRNAs, Figure 2B shows the read counts contributing to each RNA class (data for all serotypes in Supplementary Figure S6). The 23 nt siRNAs of coding genes are the most abundant ones.

We wanted to evaluate whether our analysis shows specifically produced sRNAs or degradation products. We observe that 23 nt sRNAs are predominant in our data. This fits to previous reports of the predominant sRNA length produced by Dcr1 in exogenous RNAi pathways (21,24,44). The biochemical properties of degradation products obtained from longer RNA molecules includes 5′-OH and 3′-phosphorylation. Our library preparation procedure omits 5′-OH and 3′-phosphorylated degradation products by 5′-phosphate and 3′-OH specific ligation steps, thus enriching for RNAseIII products. We know from recent research that in mammals, many RNA species produce sRNAs which are not degradation products but have regulatory power being loaded into argonautes. snoRNAs for instance produce Dicer dependent sRNAs with miRNA like functions (45). Also increasing evidence identifies more tRNA fragments produced in Dicer dependent and independent fashion (reviewed in (46)). Here, our data shows an interesting additional aspect in *Paramecium*, because Supplementary Figure S6 also shows a preference for small 23 nt antisense siRNA suggesting that RDR activity, which is not known in mammals, contributes to the accumulation of these siRNAs providing a substrate for Dicer. These data suggest that many sRNAs described here are not degradation products but result from specific or spurious RDR and Dicer activity.

As mentioned above, the rDNA cluster produces small RNAs as well. Supplementary Figure S7 shows, that small RNAs map to the entire transcribed region giving rise to the pre-rRNA. The regions of the processed rRNAs are mainly covered by sense small RNAs of more or less undefined length indicating that these are degradation products of the rRNAs (the predominant read length of 25S mapping small RNAs results from massive accumulation of only two 23-mers indicated by open arrows). Interestingly, those regions which become eliminated from the polycistronic pre-rRNA, the ETS and ITS1 and ITS2 regions, accumulate 23 nt antisense RNAs. We know from yeast, that rRNA maturation involves co-translational endonucleolytic cleavage and highly concerted trimming events to subsequently process the final rRNAs (47). Our data here suggest that these elimination processes are associated with RDR activity and antisense siRNAs in *Paramecium*.

### General properties of GSRCs

Figure 2A shows, the largest number of SRCs overlapped protein coding genes. Adding up all genes that are found to overlap a SRC in any of the four serotypes 1324 out of 40 460 protein coding genes (∼3%) are associated with SRCs. We characterized the presence of a gene overlapped by an SRC in the different serotypes using set intersection plots in Figure 2D (48). The 973 SRCs could be identified in all four serotypes, indicating absence of simple on/off mechanisms of genes for siRNA production. A clustering analysis revealed that SRCs alter their siRNA level and produce siRNAs at different abundances in a serotype-specific manner (Supplementary Figure S8). This large class of genes that is consistently overlapped by small RNAs in our WT samples is denoted as Genes associated with SRCs (GSRCs for short). These 973 GSRCs are the set of genes that are clearly overlapped by SRCs and non-specific small base overlaps have been removed (see ‘Materials and Methods’ section), because they are later used for comparison with mRNA sequencing data. Among the GSRCs, there are 78 genes involved in developmental regulation during autogamy according to (49) and also 10 heat shock proteins. One of these, HSP70PT1, was described to be one of the most regulated cytosolic proteins during heat shock response (27).

#### Analysis of biological functions

We conducted GO enrichment using the Ontologizer software (see ‘Materials and Methods’ section) on the genes that were overlapped by SRCs for each serotype, to investigate enriched functions. We found many different biological processes to be enriched in these genes including e.g.*translation, structural molecule activity, cellular biosynthetic process* and *gene expression* (Supplementary Table S2). Therefore siRNA regulation seems to be involved in a diverse set of pathways.

### siRNA accumulation in GSRCs is not necessarily associated with gene silencing

To examine the relationship between siRNAs and gene expression we integrated the sRNA data with mRNA expression data obtained from the same serotype samples (27). Figure 2E shows a box plot of expression values for the GSRCs compared to other genes in the genome. GSRCs showed a statistically significant higher expression (Wilcoxon test, *P* < 0.05) in all four serotypes. Similarly, we found that the expression variance is higher for the GSRCs in all serotypes (Supplementary Figure S9). Although this behavior would suggest an involvement of the siRNAs in regulation of these genes, our first insight into the siRNA/mRNA relationship of individual GSRCs data does not support a clear silencing function: many GSRCs show high mRNA levels and high siRNA levels. As this is in contrast to reports of other species, e.g. *C. elegans*, this raises the next logical question on the regulatory role of these siRNAs.

### mRNAs are predominantly the source of sRNAs in GSRCs

We wanted to investigate whether mRNAs act as the source of siRNAs for the GSRCs. Of the 973 GSRCs, 708 GSRCs had at least one EEJ. Figure 2F shows a box plot of the total sRNA read counts for GSRCs in the EEJs, and introns. There are no sRNA reads found in introns, except for the 51H serotype. In all the serotypes, we see a higher number of sRNA reads in EEJs compared to introns, which are statistically significant (Wilcoxon test, *P* < 0.05). Hence, we conclude that mRNAs act as the predominant source of sRNAs in our GSRCs.

To gain further insight into the association between siRNAs and genes, we investigated the siRNA coverage of gene regions for all GSRCs (Supplementary Table S1). Figure 2G shows a histogram of the number of bps covered by siRNAs in the GSRCs. A large fraction of GSRCs are covered over the complete gene length with siRNAs (overlap close to 100%).

For further analysis we split the GSRCs in three equally sized classes as visualized in Figure 2G: (i) genes showing only a discrete/small region covered with siRNAs (cov ≤ 46%), (ii) genes partially covered (46% > cov ≤ 88%) and (iii) those fully covered with siRNAs (cov > 88%). The three classes contain 329, 320 and 324 out of 973 GSRCs, respectively.

It is noteworthy that many genes are covered to a large extent with sense and antisense siRNAs (See Supplementary Figure S10). This suggests that RDR activity on the expressed mRNAs generate Dicer substrates for siRNA biogenesis. Thus far siRNA generation from mRNA templates was described to occur only in response to exogenous dsRNA in *Paramecium* (23). Our data imply that components of the exogenous dsRNA pathway attack endogenous mRNAs as well.

### *P. tetraurelia* contains phasing pathways

**Figure 3.**
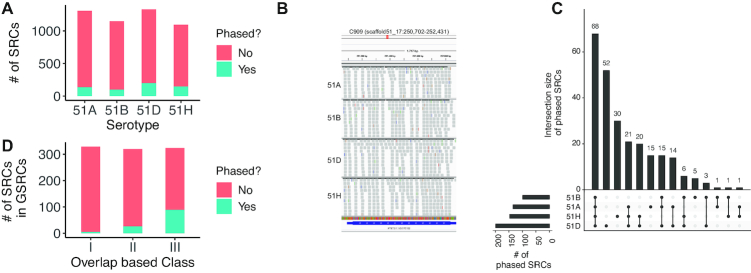
(**A**) Total number of phased clusters observed in all WT serotypes. (**B**) Example IGV screenshot of identified phased cluster, C909, annotated as a gene (ID: PTET.51.1.G0170152). (**C**) Set intersection plots for phased SRCs identified in all WT serotypes. (**D**) Number of phased clusters in each class of GSRC (as explained in Figure 2).

As many GSRCs overlap with siRNAs to a large percentage, we were wondering about possible mechanisms. We used the *P*-*score* method (see ‘Materials and Methods’ section) to further investigate whether the large amount of small RNAs is produced through RNA phasing, a mechanism currently not reported for Paramecium. Indeed, we found many regions that are phased (Supplementary Table S1). We used down-sampled read libraries to be able to compare between serotypes. The number of phased SRCs differed between serotypes (Figure 3A), where 51D had the largest number of phased SRCs. Figure 3B shows an example region of phased RNAs found in all four serotypes. An overlap analysis revealed that, while many of the SRCs are phased in all four serotypes, the majority of phased SRCs are found in only a subset of them, arguing for a serotype-specific function of phased clusters. The most unique phased SRCs were found in 51D and 51H (Figure 3C).

Phasing occurred more prevalently in class III GSRCs (Hypergeometric test *P*-value: 1*e* − 22) as illustrated in Figure 3D, suggesting that a large number of genes indeed produce siRNAs from both strands over the complete open reading frame. This suggests that RDRs act on mRNAs to produce long dsRNA as Dicer substrates.

### Many phased SRCs depend on both, RDR1 and RDR2

**Figure 4.**
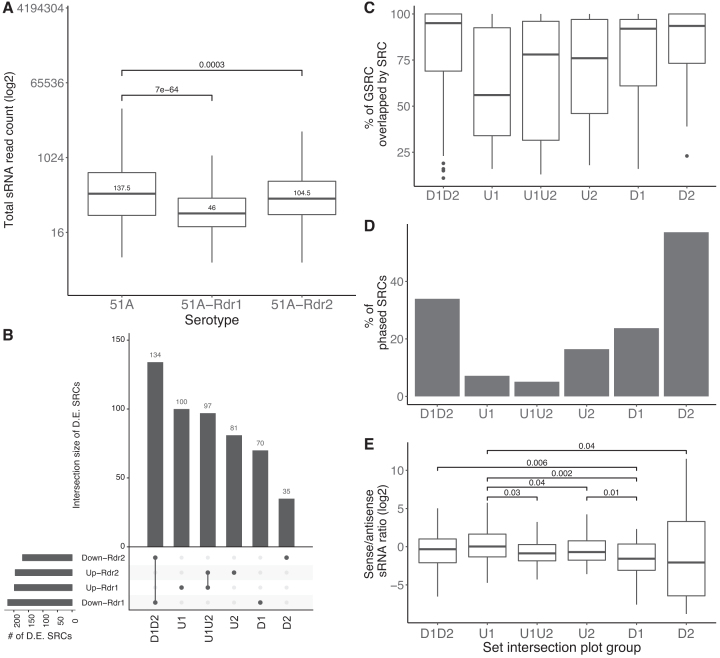
(**A**) Box plots of the total small RNA read counts (*y*-axis; log2) from the WT, and mutant samples (Mutants: 51A-Rdr1, and 51A-Rdr2). The *P*-values indicated are from two-tailed wilcoxon test. (**B**) Set intersection plots for Differentially Expressed (D.E.) SRCs identified by DESeq2 in the mutant samples. We name the intersection groups as shown here (D - Downregulated, U - Upregulated, 1, 2 represents the mutant 51A-Rdr1 and 51A-Rdr2, respectively). For these intersection groups, we show the overlap percentage of GSRC by SRCs (**C**), the percentage of phased SRCs (**D**) and the sense/antisense ratio (*y*-axis; log2) in WT (**E**) here. In (E) the *P*-values (one-tailed wilcoxon test) are indicated for only groups with statistically significant differences.

After characterization of the SRCs, we decided to manipulate the system and obtained sRNA and mRNA sequencing data for RDR1, and RDR2 mutants (see ‘Materials and Methods’ section) in serotype 51A. Both mutant lines are derived from forward genetic mutagenesis screens to obtain genes involved in dsRNA feeding (16). Figure 4A shows the normalized siRNA read counts for all SRCs in the WT, and mutant samples. A statistically significant (two-tailed Wilcoxon test) reduction of siRNA reads in many SRCs is observed in both RDR mutants. As RDR1 and RDR2 were extensively described for their action of exogenous dsRNA being responsible for primary (1^*o*^) siRNA accumulation, these data indicate that they are not exclusively involved in exogenous RNAi but also in endogenous RNAi. Thus, their action is not limited into the recognition of self- and non-self RNA but RDR activity acts on both endo- and exogenous RNAs. We used the RAPID pipeline (30) to perform a differential expression analysis for all SRCs making use of our replicate data (see ‘Materials and Methods’ section). Analysis of differentially expressed SRCs in Figure 4B revealed that the majority of differentially downregulated SRCs depend on both RDRs. This is again similar to the 1° feeding siRNAs, where accumulation is dependent on both RDRs (15,23). Although it is unclear why two RDRs are necessary to produce siRNAs from dsRNA which is exogenously supplied and taken up from the food from dsRNA producing bacteria, the same appears to be true for a lot of endogenous siRNA producing loci. Figure 4B also indicates that several SRCs are upregulated. This could be due to general secondary or indirect effects, because we also observed that RDR mutants induce massive transcriptomic alterations (Supplementary Figure S10).

In order to compare these different SRC groups we named them after their occurrence in mutants data. For example the D1D2 group refers to SRCs that are downregulated in both RDR1 and RDR2 mutants. Similarly U1U2 denotes SRCs that are upregulated in both mutants. We further investigated the different intersection groups (shown in Figure 4B), and their GSRC characteristics in WT (51A) samples. Figure 4C shows that GSRCs that overlap with downregulated SRCs (D1D2, D1, D2) are often fully covered by siRNAs and contain the largest percentage of SRCs that are phased (Figure 4D) in WT. We thus conclude that RDRs are predominantly involved in phased SRCs covering the entire ORF. *Vice versa*, one can conclude that many full length mRNAs are converted into siRNAs by RDRs. This is clearly different in the up-regulated categories U1/U2, U1U2, therefore suggesting that the siRNA accumulation mechanism of these loci is different. The D1 and D2 SRCs showed a stronger antisense bias compared to other categories. D1D2 category SRCs are downregulated in both mutants (Figure 4E), suggesting that small RNA production in these SRCs differs as well. In contrast, SRCs that are upregulated (U1, U2, U1U2 categories) only overlap with few GSRCs that are phased and are covered less with siRNAs (Figure 4C and D). SRCs from the U1 category are the only ones that show a small sense siRNA bias, all others have an antisense bias (Figure 4E). It seems likely that the up-regulated SRCs have indeed different genetic properties or requirements compared to down-regulated RDR-dependent SRCs.

**Figure 5.**
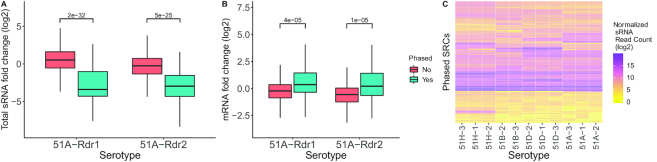
(**A**) Boxplots of total sRNA fold change (*y*-axis; log2 mutant/WT) in each mutant is shown. Boxplots are grouped based on whether a SRC is predicted as an unphased, or phased loci. (**B**) Same as (A), but shows the mRNA fold change (*y*-axis; log2 mutant/WT). The *P*-values indicated (A and B) are from two-tailed wilcoxon test. (**C**) A heatmap of all the phased SRCs, total small RNA read counts (log2) of all the WT replicates is shown.

In order to investigate the effect of RDR mutants genome wide, we looked at the fold changes of total sRNA accumulation (Figure 5A), and mRNA expression (Figure 5B). In both RDR mutants, the sRNA fold change of phased SRCs is statistically significantly lower than unphased. This suggests that phased SRCs have different genetic requirements and thus different siRNA accumulation mechanisms than others. Integrating the transcriptome data of the mutants, we observe that phased siRNAs are indeed acting negatively *in cis* as phased clusters produce more mRNA in RDR mutants (Figure 5B). This is not the case for the non phased SRCs, which raises the question of how they accumulate, and most importantly the function of their siRNAs. We conclude that phasing is a prevalent mechanism in *Paramecium* occurring in many different regions in the genome. These phased regions are affected in two mutants, of the known RNAi enzymes; RDR1 and RDR2, and are evidently negatively correlated with gene expression. Figure 5C shows a clustered heat map of phased SRCs, which clearly distinguishes the variability of phased SRCs across different serotypes. We can therefore conclude that the phased SRCs contribute to the WT’s transcriptome dynamics. For the interpretation of RDR mutants we need to consider secondary effects: the massive transcriptomic changes also include RNAi components and many, e.g. Ptiwi12, 13 and 14 (Supplementary Table S1), show downregulation in RDR mutant lines. Although the Ptiwi downregulation could be the result of a diminished sRNA abundance, we cannot directly relate the loss of RDR activity to a reduction of small RNAs for all clusters.

### Positive and negative correlation of siRNAs with gene expression

**Figure 6.**
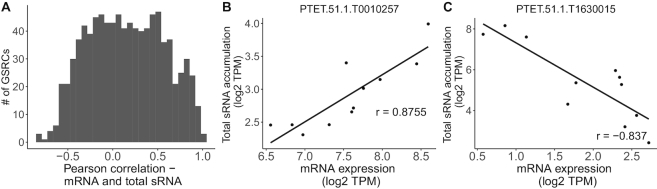
(**A**) A histogram of the number of GSRCs (*y*-axis) plotted against the Pearson correlation of mRNA expression and total sRNA (*x*-axis) of all WT replicates. Scatter plots of an example GSRC with high positive (**B**) and negative (**C**) correlation with mRNA expression (see r values in the plot). (*x*-axis; log2 TPM) against total sRNA accumulation (*y*-axis; log2 TPM) are shown.

Next to the source of the siRNAs, we also spent attention to their target, especially as we did not observe strict silencing in the originating GSRCs. Thus, we were wondering about the possible function of those siRNAs that overlap the 973 GSRCs. We therefore produced replicate data of sRNA-seq and mRNA-seq from the same serotype samples for all four serotypes with three replicates each. First, we checked whether the mRNA expression level of the gene shows an association to the siRNA abundance in that same gene. Figure 6A shows the histogram of gene correlation values computed for siRNA against mRNA expression over all paired replicate samples. We assessed the significance of those Pearson correlation values and corrected for multiple testing using Benjamini–Hochberg method (50). We found that ∼8% were statistically significant (FDR < 0.05), with 71 and 3 GSRCs showing positive and negative correlation, respectively. Surprisingly, no clear trend emerged and we saw genes, where siRNA abundance was both positively, and negatively correlated with gene expression. Figure 6B and C show two gene examples (T0010257 and T1630015) with statistically significant correlation, that illustrate these different behaviors. This analysis reveals that these siRNAs are probably involved in a complex regulatory machinery, which will be probed in the future.

## DISCUSSION

In this study, we made a first description of genome-wide sRNAs in *Paramecium* in order to understand their function and to gain insight into their accumulation pathways. We have designed a pipeline to combine small RNA read data from four different serotypes. One important aspect in this was the adjustment of sRNA clusters that overlap genes and correct for spurious overlaps at gene boundaries. Our analyses defined a large set of SRCs of which most of them exist in all serotypes. However, sRNA accumulation clearly dissects the four serotypes thus creating specific sRNA patterns. ShortStack screens for miRNA precursors *de novo* using RNA folding and several filtering steps to ensure there are no false positives. We did not identify any canonical miRNAs, unlike in plants and animals where miRNAs occur ubiquitously. In fungi, miRNA-like loci have been identified (51,52). We therefore conclude that for the majority of SRCs described here RDR activity produces a dsRNA Dicer substrate.

### SRCs in protein coding genes

We have identified ∼1300 genes associated with sRNAs. A direct comparison of our data to other organisms is difficult. The decision of whether a gene produces small RNAs or not strongly depends on the sequencing depth and the individual thresholds to predict a cluster. In *C. elegans* for instance ∼250 genes are believed to be regulated by an individual RNAi pathway, the associated siRNAs are antisense and the above mentioned genes are up-regulated in RNAi mutants (19). Here, the situation appears to be more complex. Some genes clearly accumulate massive amounts of sense siRNAs, and we need to investigate their function as it seems unlikely that these siRNAs are stabilized by chance. This is also different to most other species, *e.g*. endogenous siRNAs in *C. elegans* are perfectly antisense (53) as well as in the closely related ciliate *Tetrahymena* (54). Here, our data do not allow us to dissect whether siRNAs have a regulatory role or not. Although they are 23 nt in length and carry a 5’-monophosphate, vouching for the involvement of Dicer in their biosynthesis, they may result from spurious Dicer activity on mRNA substrates. Thus, we cannot exclude the possibility that some highly expressed mRNA species accidentally recruit the RNAi machinery, which we observe in our data as the positively correlated clusters. However, we were only able to analyze sRNA function *in cis*, meaning on the originating gene, where we cannot see a negative correlation for many GSRCs. Maybe, the fact that most of these GSRCs stabilize the sense strand of the siRNAs may be a hint that the target of the siRNAs should not be the originating mRNA. These stabilized sense siRNAs may be involved *in trans* silencing of other genes or loci in the genome. As this was shown to occur by transgene-induced silencing in *Paramecium* in which siRNA producing transgenes can silence homologous remote loci *in trans* (24,55). This may also be an important mechanism for concerted endogenous gene regulation. Similarly, trans regulation was also suggested for the multigene family of surface antigens (56) and indeed, these genes are among the SRCs. In contrast to other ciliates and other species, *Paramecium* may have evolved additional mechanisms to regulate gene expression by homology, because of the high number of gene duplicates due to three successive genome duplications (41).

### RDR1 and RDR2 produce phased siRNAs from exogenous and endogenous substrates

Among these SRCs, we find phasing signatures for a subset. The subsequent analyses identified three major criteria for these: (i) they are enriched in genes showing full siRNA coverage along the orf, (ii) most of them depend on RDR1 and RDR2, and (iii) the corresponding genes show larger expression variability in the WT. It seems surprising that the phased SRCs depend on these two RDRs. We have described the same dependency for 1^*o*^ siRNAs produced from exogenous dsRNA, although it is still not understood why RDRs are necessary for dicing the dsRNA (15). However, our data indicate that RDR1 and RDR2 also act together on endogenous mRNAs, not limiting their function to the recognition of exogenous RNA. The absence of siRNA strand bias, the tendency for full coverage of mRNA orfs and their dependency on RDR1/2 are criteria reminiscent of 1^*o*^ siRNAs from exogenous RNA. The fourth criteria connecting them is the silencing capacity. In *Paramecium*, the silencing phenotype was shown to be correlated with the abundance of 1^*o*^ siRNAs, not 2^*o*^ siRNAs as in *C. elegans*. We observed the same for the phased clusters in this study, because their mRNA levels are clearly increased in the two mutant lines. Analysis of WT transcriptomes revealed that the phased clusters differ, thus we conclude that RDR1/2 mediated siRNA accumulation in phased clusters contributes to transcriptome alterations and represents an endogenous gene regulation mechanism.

In many instances, species increased the number of genes for RNAi components, e.g. by gene duplication and gained specialization performing individual pathways and processes as shown, e.g. in plants and flies, which evolved distinct Dicers for endo- and exogenous RNAi (57). As *Paramecium* owns four RDR genes, this overlap between exo- and endogenous RNAi, not only in the components but also in the combination of them, is surprising. Next to the similarities of the exogenous feeding pathways between *C. elegans* and *Paramecium*, we cannot be sure whether this mechanism represents antiviral pathways, because the laboratory strains of both species do not allow for testing of native viruses. At least for *C. elegans* there is experience with an individual virus triggering 1^*o*^ and 2^*o*^ siRNAs (58), reviewed in (59).

In the exogenous RNAi pathways, we do not know the function of 2^*o*^ siRNAs in *Paramecium*: we know that they are less abundant than 1^*o*^ siRNAs and 5’-monophosphorylated, which clearly distinguishes them from 2^*o*^ siRNAs from the nematode feeding pathway. In *Paramecium*, only a few genes have been analyzed for their feeding induced siRNAs, which do not seem to be associated with mRNA degradation but rather be a subsequent event of 1^*o*^ siRNA attack. If this is true for some endogenous clusters, 2^*o*^ siRNAs could explain SRCs which are positively correlated with mRNA expression. If these are attacked by individual 1^*o*^ siRNAs, the amount of 2^*o*^ siRNA accumulation could be a function of mRNA abundance, thus revealing a positive correlation between mRNA and siRNAs.

In summary, phased SRCs show features of plant secondary siRNAs, where RDRs attack mRNAs to produce a double stranded Dicer substrate. The question raises, why these mRNAs are RDR substrates. On the one hand they could be attacked by individual siRNAs. On the other hand these could be RNAi independent as a previous study of *Tetrahymena* indicated seven phased siRNA loci and suggested that endonucleolytic cleavage of mRNAs generates a fraction of non poly-adenylated RNA for access to RDR and Dicer (60,61). A similar mechanism could allow RDR1/2 to access mRNAs in *Paramecium*. This suggests a general role of the complex of these two RDRs, which is likely the dissection of Dicer substrates. If dsRNA is supplied to *Paramecium*, RDR1 and RDR2 are necessary to create Dicer products (15). Likely, they amplify the dsRNA to provide a suitable substrate as e.g. a Dicer protein from the related *Tetrahymena* was shown to require a 5’-triphosphate for discrete siRNA cleavage (62). Thus, their function may not be amplification but initiation. The fact that 2^*o*^ siRNAs are indeed less abundant than 1^*o*^ siRNAs in *Paramecium* (23,24) suggests that this single cell organism might not need to amplify silencing signals for distribution to other cells and tissues, which is different to other multicellular organisms.

### SRCs in non-coding RNA loci

We identified many SRCs in loci encoding regulatory RNAs such as snRNA, snoRNA and tRNA. This raises the questions whether we detect degradation products or specifically accumulated sRNAs. Degradation products of longer RNAs contain partly different biochemical properties such as 5’-OH and 3’-phosphates, which our library preparation procedure will exclude. Also, these SRCs show predominantly 23 nt read length, which is the length preferred by *Paramecium*’s Dcr1 as shown for several pathways (15,21). In mammals, fragments of snRNA, snoRNA and tRNAs are known to be produced in a Dicer dependent manner from secondary structures. An increasing number of publications indicate that these small RNAs are not solely results of spurious Dicer activity, but the produced small RNAs can have regulatory power (e.g. (63,64)). Our data indicate that the situation is different in *Paramecium*: RDR activity, which is believed to be absent in mammals, appears to be necessary to produce sRNAs from these longer RNAs and as a result we can see the 23 nt antisense strand in the resulting SRC. This would indicate a fundamental mechanistic difference in the biogenesis of these sRNA. The ribosomal small RNAs and their distribution observed over the rDNA cluster appears different to a recent report from *C. elegans*, where antisense siRNAs of the 18S and 26S rRNAs downregulate pre-rRNA (65). The predominant accumulation of antisense siRNAs in eliminated regions suggests that these are associated with the elimination process itself rather than a regulation of the entire pre-rRNA.

We also find few sRNAs in the intergenic regions. A possibility for their function would be an involvement in replication and regulation of polyploidy. In the ciliate *Oxytricha*, Dicer and RDR-dependent chromatin associated siRNAs have recently been shown to control DNA copy number possibly via control of DNA replication (66). However, in comparison to large, up to 1 MB chromosomes in *Paramecium, Oxytricha* macronuclei contain gene sized chromosomes containing a single gene only (67). As such, siRNA mediated control of chromosome copy number would affect many more genes in *Paramecium*. However, Garnier *et al.* reported the influence of exogenous siRNAs on the DNA copy number of homologous genes in sexual progeny, because by activation of several telomere addition sites individual genetic loci can be prevented from developmental amplification (68) contributing to a general heterogeneity of macronuclear chromosomes (69). In addition, it was shown that chromosome fragmentation and telomere addition, not necessarily accompanied by copy number variations, can influence gene expression (56). Further studies need to be conducted whether the intergenic SRCs could be involved in such processes.

## CONCLUSION

We described here the first genome-wide profiling of small RNAs during vegetative growth of *Paramecium*. We cannot identify any miRNAs, but we identify many SRCs in protein coding genes. Our data suggest that the *Paramecium* RNAi machinery, including RDR activity, produces siRNAs against all analyzed RNA classes. Especially for a subset of protein coding genes, our data shows ORF-wide phasing implying efficient conversion of mRNA into dsRNA and subsequent stabilization of Dicer cleavage products. In contrast to other organisms, siRNA accumulation of protein coding genes is not strictly strand biased and not exclusively associated with gene silencing *in cis*. The resulting genome wide SRC pattern are therefore highly specific and our quantitative sRNA analysis allows us to distinguish between different transcriptomic states/serotypes and therefore the SRCs are altered by the environmental circumstances, e.g. temperature.

## DATA AVAILABILITY

The datasets created as part of this study can be accessed at ENA (Accession: PRJEB25903).

## Supplementary Material

gkz553_Supplemental_Files

## Notes

Present address: Vidya Oruganti, Department of Plant Developmental Biology, Max Planck Institute for Plant Breeding Research, 50829 Cologne, Germany.

## References

[1] Carthew R.W., Sontheimer E.J. Origins and mechanisms of miRNAs and siRNAs. Cell. 2009; 136:642–655. 19239886 10.1016/j.cell.2009.01.035 PMC2675692

[2] Allen E., Xie Z., Gustafson A.M., Carrington J.C. microRNA-directed phasing during trans-acting siRNA biogenesis in plants. Cell. 2005; 121:207–221. 15851028 10.1016/j.cell.2005.04.004

[3] Ha M., Kim V.N. Regulation of microRNA biogenesis. Nat. Rev. Mol. Cell Biol. 2014; 15:509–524. 25027649 10.1038/nrm3838

[4] Pinzón N., Bertrand S., Subirana L., Busseau I., Escrivá H., Seitz H. Functional lability of RNA-dependent RNA polymerases in animals. PLoS Genet. 2019; 15:e1007915. 30779744 10.1371/journal.pgen.1007915 PMC6396948

[5] Li Y., Lu J., Han Y., Fan X., Ding S.-W. RNA interference functions as an antiviral immunity mechanism in mammals. Science. 2013; 342:231–234. 24115437 10.1126/science.1241911 PMC3875315

[6] Maillard P., Ciaudo C., Marchais A., Li Y., Jay F., Ding S., Voinnet O. Antiviral RNA interference in mammalian cells. Science. 2013; 342:235–238. 24115438 10.1126/science.1241930 PMC3853215

[7] Maillard P.V., Van der Veen A.G., Deddouche-Grass S., Rogers N.C., Merits A., e Sousa C.R. Inactivation of the type I interferon pathway reveals long double-stranded RNA-mediated RNA interference in mammalian cells. EMBO J. 2016; 35:2505–2518. 27815315 10.15252/embj.201695086 PMC5167344

[8] Parent J.-S., de Alba M., Emilio A., Vaucheret H. The origin and effect of small RNA signaling in plants. Front. Plant Sci. 2012; 3:179. 22908024 10.3389/fpls.2012.00179 PMC3414853

[9] Fei Q., Xia R., Meyers B.C. Phased, secondary, small interfering RNAs in posttranscriptional regulatory networks. Plant Cell. 2013; 25:2400–2415. 23881411 10.1105/tpc.113.114652 PMC3753373

[10] Pak J., Fire A. Distinct populations of primary and secondary effectors during RNAi in C. elegans. Science. 2007; 315:241–244. 17124291 10.1126/science.1132839

[11] Sijen T., Steiner F.A., Thijssen K.L., Plasterk R.H. Secondary siRNAs result from unprimed RNA synthesis and form a distinct class. Science. 2007; 315:244–247. 17158288 10.1126/science.1136699

[12] Ashe A., Bélicard T., Le Pen J., Sarkies P., Frézal L., Lehrbach N.J., Félix M.-A., Miska E.A. A deletion polymorphism in the Caenorhabditis elegans RIG-I homolog disables viral RNA dicing and antiviral immunity. Elife. 2013; 2:e00994. 24137537 10.7554/eLife.00994 PMC3793227

[13] Gent J.I., Lamm A.T., Pavelec D.M., Maniar J.M., Parameswaran P., Tao L., Kennedy S., Fire A.Z. Distinct phases of siRNA synthesis in an endogenous RNAi pathway in C. elegans soma. Mol. Cell. 2010; 37:679–689. 20116306 10.1016/j.molcel.2010.01.012 PMC2838994

[14] Maniar J.M., Fire A.Z. EGO-1, a C. elegans RdRP, modulates gene expression via production of mRNA-templated short antisense RNAs. Curr. Biol. 2011; 21:449–459. 21396820 10.1016/j.cub.2011.02.019 PMC3073447

[15] Marker S., Le Mouel A., Meyer E., Simon M. Distinct RNA-dependent RNA polymerases are required for RNAi triggered by double-stranded RNA versus truncated transgenes in Paramecium tetraurelia. Nucleic Acids Res. 2010; 38:4092–4107. 20200046 10.1093/nar/gkq131 PMC2896523

[16] Marker S., Carradec Q., Tanty V., Arnaiz O., Meyer E. A forward genetic screen reveals essential and non-essential RNAi factors in Paramecium tetraurelia. Nucleic Acids Res. 2014; 42:7268–7280. 24860163 10.1093/nar/gku223 PMC4066745

[17] Simon M., Plattner H. Unicellular eukaryotes as models in cell and molecular biology: critical appraisal of their past and future value. Int. Rev. Cell Mol. Biol. 2014; 309:141–198. 24529723 10.1016/B978-0-12-800255-1.00003-X

[18] Pilling O.A., Rogers A.J., Gulla-Devaney B., Katz L.A. Insights into transgenerational epigenetics from studies of ciliates. Eur. J. Protistol. 2017; 61:366–375. 28689743 10.1016/j.ejop.2017.05.004 PMC5827946

[19] Grishok A., Hoersch S., Sharp P.A. RNA interference and retinoblastoma-related genes are required for repression of endogenous siRNA targets in Caenorhabditis elegans. Proc. Natl. Acad. Sci. U.S.A. 2008; 105:20386–20391. 19073934 10.1073/pnas.0810589105 PMC2629315

[20] Mansisidor A.R., Cecere G., Hoersch S., Jensen M.B., Kawli T., Kennedy L.M., Chavez V., Tan M.W., Lieb J.D., Grishok A. A conserved PHD finger protein and endogenous RNAi modulate insulin signaling in Caenorhabditis elegans. PLoS Genet. 2011; 7:e1002299. 21980302 10.1371/journal.pgen.1002299 PMC3183084

[21] Lepere G., Nowacki M., Serrano V., Gout J.-F., Guglielmi G., Duharcourt S., Meyer E. Silencing-associated and meiosis-specific small RNA pathways in Paramecium tetraurelia. Nucleic Acids Res. 2008; 37:903–915. 19103667 10.1093/nar/gkn1018 PMC2647294

[22] Sandoval P.Y., Swart E.C., Arambasic M., Nowacki M. Functional diversification of Dicer-like proteins and small RNAs required for genome sculpting. Dev. Cell. 2014; 28:174–188. 24439910 10.1016/j.devcel.2013.12.010

[23] Carradec Q., Götz U., Arnaiz O., Pouch J., Simon M., Meyer E., Marker S. Primary and secondary siRNA synthesis triggered by RNAs from food bacteria in the ciliate Paramecium tetraurelia. Nucleic Acids Res. 2015; 43:1818–1833. 25593325 10.1093/nar/gku1331 PMC4330347

[24] Götz U., Marker S., Cheaib M., Andresen K., Shrestha S., Durai D.A., Nordström K.J., Schulz M.H., Simon M. Two sets of RNAi components are required for heterochromatin formation in trans triggered by truncated transgenes. Nucleic Acids Res. 2016; 44:5908–5923. 27085807 10.1093/nar/gkw267 PMC4937312

[25] Simon M.C., Marker S., Schmidt H.J. Inefficient serotype knock down leads to stable coexistence of different surface antigens on the outer membrane in Paramecium tetraurelia. Eur. J. Protistol. 2006; 42:49–53. 17070750 10.1016/j.ejop.2005.09.003

[26] Martin M. Cutadapt removes adapter sequences from high-throughput sequencing reads. EMBnet. journal. 2011; 17:10–12.

[27] Cheaib M., Dehghani Amirabad A., Nordström K.J., Schulz M.H., Simon M. Epigenetic regulation of serotype expression antagonizes transcriptome dynamics in Paramecium tetraurelia. DNA Res. 2015; 22:293–305. 26231545 10.1093/dnares/dsv014 PMC4535620

[28] Johnson N.R., Yeoh J.M., Coruh C., Axtell M.J. Improved placement of multi-mapping small RNAs. G3. 2016; 6:2103–2111. 27175019 10.1534/g3.116.030452 PMC4938663

[29] Quinlan A.R., Hall I.M. BEDTools: a flexible suite of utilities for comparing genomic features. Bioinformatics. 2010; 26:841–842. 20110278 10.1093/bioinformatics/btq033 PMC2832824

[30] Karunanithi S., Simon M., Schulz M.H. Automated analysis of small RNA datasets with RAPID. PeerJ. 2019; 7:e6710. 30993044 10.7717/peerj.6710 PMC6462184

[31] Love M.I., Huber W., Anders S. Moderated estimation of fold change and dispersion for RNA-seq data with DESeq2. Genome Biol. 2014; 15:550. 25516281 10.1186/s13059-014-0550-8 PMC4302049

[32] Robinson J.T., Thorvaldsdóttir H., Winckler W., Guttman M., Lander E.S., Getz G., Mesirov J.P. Integrative genomics viewer. Nat. Biotechnol. 2011; 29:24–26. 21221095 10.1038/nbt.1754 PMC3346182

[33] Patro R., Duggal G., Love M.I., Irizarry R.A., Kingsford C. Salmon provides fast and bias-aware quantification of transcript expression. Nat. Methods. 2017; 14:417–419. 28263959 10.1038/nmeth.4197 PMC5600148

[34] Arnaiz O., Cain S., Cohen J., Sperling L. ParameciumDB: a community resource that integrates the Paramecium tetraurelia genome sequence with genetic data. Nucleic Acids Res. 2007; 35:D439–D444. 17142227 10.1093/nar/gkl777 PMC1669747

[35] Howell M.D., Fahlgren N., Chapman E.J., Cumbie J.S., Sullivan C.M., Givan S.A., Kasschau K.D., Carrington J.C. Genome-wide analysis of the RNA-DEPENDENT RNA POLYMERASE6/DICER-LIKE4 Pathway in Arabidopsis Reveals Dependency on miRNA- and tasiRNA-directed targeting. Plant Cell. 2007; 19:926–942. 17400893 10.1105/tpc.107.050062 PMC1867363

[36] Preer L.B., Rudman B., Pollack S., Preer J.R. Does ribosomal DNA get out of the micronuclear chromosome in Paramecium tetraurelia by means of a rolling circle. Mol. Cell Biol. 1999; 19:7792–7800. 10523668 10.1128/mcb.19.11.7792 PMC84842

[37] Barth D., Krenek S., Fokin S.I., Berendonk T.U. Intraspecific genetic variation in Paramecium revealed by mitochondrial cytochrome C oxidase I sequences. J. Eukaryot. Microbiol. 2006; 53:20–25. 16441579 10.1111/j.1550-7408.2005.00068.x

[38] Zhang Z., Carriero N., Zheng D., Gerstein M., Karro J., Harrison P.M. PseudoPipe: an automated pseudogene identification pipeline. Bioinformatics. 2006; 22:1437–1439. 16574694 10.1093/bioinformatics/btl116

[39] Bauer S., Grossmann S., Vingron M., Robinson P.N. Ontologizer 2.0:a multifunctional tool for GO term enrichment analysis and data exploration. Bioinformatics. 2008; 24:1650–1651. 18511468 10.1093/bioinformatics/btn250

[40] Simon M.C., Schmidt H.J. Antigenic variation in ciliates: antigen structure, function, expression. J. Eukaryot. Microbiol. 2007; 54:1–7. 17300509 10.1111/j.1550-7408.2006.00226.x

[41] Aury J.M., Jaillon O., Duret L., Noel B., Jubin C., Porcel B.M., Segurens B., Daubin V., Anthouard V., Aiach N. et al. Global trends of whole-genome duplications revealed by the ciliate Paramecium tetraurelia. Nature. 2006; 444:171–178. 17086204 10.1038/nature05230

[42] Arnaiz O., Sperling L. ParameciumDB in 2011: new tools and new data for functional and comparative genomics of the model ciliate Paramecium tetraurelia. Nucleic Acids Res. 2011; 39:D632–D636. 20952411 10.1093/nar/gkq918 PMC3013783

[43] de Vanssay A., Sallet E., Van Dijk E., Gouzy J., Sperling L., Lhuillier-Akakpo M., Bétermier M., Arnaiz O., Duharcourt S. Improved methods and resources for paramecium genomics: transcription units, gene annotation and gene expression. BMC Genomics. 2017; 18:483. 28651633 10.1186/s12864-017-3887-z PMC5485702

[44] Marker S., Carradec Q., Tanty V., Arnaiz O., Meyer E. A forward genetic screen reveals essential and non-essential RNAi factors in Paramecium tetraurelia. Nucleic Acids Res. 2014; 42:7268–7280. 24860163 10.1093/nar/gku223 PMC4066745

[45] Falaleeva M., Stamm S. Processing of snoRNAs as a new source of regulatory non-coding RNAs: snoRNA fragments form a new class of functional RNAs. Bioessays. 2013; 35:46–54. 23180440 10.1002/bies.201200117 PMC3732821

[46] Sun C., Fu Z., Wang S., Li J., Li Y., Zhang Y., Yang F., Chu J., Wu H., Huang X. et al. Roles of tRNA-derived fragments in human cancers. Cancer Lett. 2018; 414:16–25. 29107107 10.1016/j.canlet.2017.10.031

[47] Henras A.K., Plisson-Chastang C., O’Donohue M.F., Chakraborty A., Gleizes P.E. An overview of pre-ribosomal RNA processing in eukaryotes. WIREs RNA. 2015; 6:225–242. 25346433 10.1002/wrna.1269 PMC4361047

[48] Conway J.R., Lex A., Gehlenborg N. UpSetR: an R package for the visualization of intersecting sets and their properties. Bioinformatics. 2017; 33:2938–2940. 28645171 10.1093/bioinformatics/btx364 PMC5870712

[49] Arnaiz O., Gout J.F., Betermier M., Bouhouche K., Cohen J., Duret L., Kapusta A., Meyer E., Sperling L. Gene expression in a paleopolyploid: a transcriptome resource for the ciliate Paramecium tetraurelia. BMC Genomics. 2010; 11:547. 20932287 10.1186/1471-2164-11-547 PMC3091696

[50] Benjamini Y., Hochberg Y. Controlling the false discovery rate: a practical and powerful approach to multiple testing. J. methodRoy. Statist. Soc. Ser. B. 1995; 57:289–300.

[51] Lau S.K., Chow W.-N., Wong A.Y., Yeung J.M., Bao J., Zhang N., Lok S., Woo P.C., Yuen K.-Y. Identification of microRNA-like RNAs in mycelial and yeast phases of the thermal dimorphic fungus Penicillium marneffei. PLoS Negl. Trop. Dis. 2013; 7:e2398. 23991243 10.1371/journal.pntd.0002398 PMC3749987

[52] Lee H.-C., Li L., Gu W., Xue Z., Crosthwaite S.K., Pertsemlidis A., Lewis Z.A., Freitag M., Selker E.U., Mello C.C. et al. Diverse pathways generate MicroRNA-like RNAs and Dicer-Independent small interfering RNAs in fungi. Mol. Cell. 2010; 38:803–814. 20417140 10.1016/j.molcel.2010.04.005 PMC2902691

[53] Grishok A. Endogenous RNAi and adaptation to environment in C. elegans. Worm. 2012; 1:129–133. 24058837 10.4161/worm.19538 PMC3670229

[54] Lee S.R., Collins K. Two classes of endogenous small RNAs in Tetrahymena thermophila. Genes Dev. 2006; 20:28–33. 16357212 10.1101/gad.1377006 PMC1356098

[55] Pirritano M., Götz U., Karunanithi S., Nordström K., Schulz M., Simon M. Environmental temperature controls accumulation of transacting siRNAs involved in heterochromatin formation. Genes. 2018; 9:117. 10.3390/genes9020117 PMC5852613 29466322

[56] Baranasic D., Oppermann T., Cheaib M., Cullum J., Schmidt H., Simon M. Genomic characterization of variable surface antigens reveals a telomere position effect as a prerequisite for RNA interference-mediated silencing in Paramecium tetraurelia. Mbio. 2014; 5:e01328-14. 25389173 10.1128/mBio.01328-14 PMC4235209

[57] Ding S.-W., Li H., Lu R., Li F., Li W.-X. RNA silencing: a conserved antiviral immunity of plants and animals. Virus Res. 2004; 102:109–115. 15068886 10.1016/j.virusres.2004.01.021

[58] Félix M.-A., Ashe A., Piffaretti J., Wu G., Nuez I., Bélicard T., Jiang Y., Zhao G., Franz C.J., Goldstein L.D. et al. Natural and experimental infection of Caenorhabditis nematodes by novel viruses related to nodaviruses. PLoS Biol. 2011; 9:e1000586. 21283608 10.1371/journal.pbio.1000586 PMC3026760

[59] Sarkies P., Miska E.A. RNAi pathways in the recognition of foreign RNA: antiviral responses and host–parasite interactions in nematodes. Biochem. Soc. Trans. 2013; 41:876–880. 23863148 10.1042/BST20130021

[60] Talsky K.B., Collins K. Strand-asymmetric endogenous Tetrahymena small RNA production requires a previously uncharacterized uridylyltransferase protein partner. RNA. 2012; 18:1553–1562. 22706992 10.1261/rna.033530.112 PMC3404375

[61] Couvillion M.T., Lee S.R., Hogstad B., Malone C.D., Tonkin L.A., Sachidanandam R., Hannon G.J., Collins K. Sequence, biogenesis, and function of diverse small RNA classes bound to the Piwi family proteins of Tetrahymena thermophila. Genes Dev. 2009; 23:2016–2032. 19656801 10.1101/gad.1821209 PMC2751968

[62] Lee S.R., Collins K. Physical and functional coupling of RNA-dependent RNA polymerase and Dicer in the biogenesis of endogenous siRNAs. Nat. Struct. Mol. Biol. 2007; 14:604–610. 17603500 10.1038/nsmb1262

[63] Anderson P., Ivanov P. tRNA fragments in human health and disease. FEBS Lett. 2014; 588:4297–4304. 25220675 10.1016/j.febslet.2014.09.001 PMC4339185

[64] Scott M.S., Ono M. From snoRNA to miRNA: dual function regulatory non-coding RNAs. Biochimie. 2011; 93:1987–1992. 21664409 10.1016/j.biochi.2011.05.026 PMC3476530

[65] Zhou X., Feng X., Mao H., Li M., Xu F., Hu K., Guang S. RdRP-synthesized antisense ribosomal siRNAs silence pre-rRNA via the nuclear RNAi pathway. Nat. Struct. Mol. Biol. 2017; 24:258–269. 28165511 10.1038/nsmb.3376

[66] Khurana J.S., Clay D., Moreira S., Wang X., Landweber L.F. Small RNA-mediated regulation of DNA dosage in the ciliate Oxytricha. RNA. 2017; 24:18–29. 29079634 10.1261/rna.061333.117 PMC5733567

[67] Swart E.C., Bracht J.R., Magrini V., Minx P., Chen X., Zhou Y., Khurana J.S., Goldman A.D., Nowacki M., Schotanus K. et al. The Oxytricha trifallax macronuclear genome: a complex eukaryotic genome with 16,000 tiny chromosomes. PLoS Biol. 2013; 11:e1001473. 23382650 10.1371/journal.pbio.1001473 PMC3558436

[68] Garnier O., Serrano V., Duharcourt S., Meyer E. RNA-mediated programming of developmental genome rearrangements in Paramecium tetraurelia. Mol. Cell Biol. 2004; 24:7370–7379. 15314149 10.1128/MCB.24.17.7370-7379.2004 PMC506981

[69] Duret L., Cohen J., Jubin C., Dessen P., Goût J.-F., Mousset S., Aury J.-M., Jaillon O., Noël B., Arnaiz O. et al. Analysis of sequence variability in the macronuclear DNA of Paramecium tetraurelia: a somatic view of the germline. Genome Res. 2008; 18:585–596. 18256234 10.1101/gr.074534.107 PMC2279246

