# Supplementary material for: Exogenous RNAi mechanisms contribute to transcriptome adaptation by phased siRNA clusters in Paramecium
Source: Nucleic Acids Res. 2019 Jun 28;47(15):8036–49. doi: 10.1093/nar/gkz553 (PMC6735861; doi:10.1093/nar/gkz553)
Supplement: gkz553_Supplemental_Files [file gkz553_supplemental_files.zip › SupplementaryMethodsAndFigures.pdf]

Exogenous RNAi mechanisms contribute to transcriptome adaptation by phased siRNA clusters in *Paramecium*

Karunanithi et. al.

May 2, 2019

1 Supplementary Methods

1.1 SRC Boundary Modifications

Before correlating sRNA accumulation with mRNA expression, we did a quick scan of SRCs in IGV Browser (version 2.3.91). This investigation of SRCs showed occurrences of non-specific boundaries.

What are non-specific boundaries?

Consider example in Supplementary Figure S2. When performing an annotation overlap (intersectBed; bedtools v2.23), of a SRC region (C1732; shown in the above figure) overlaps with three genes. Whereas, only one gene is responsible for the SRC in the example, two neighboring genes got included in the overlap as well. This is due to the expansion of the SRC localization, which could have be due to one or more of the following reasons: (i) Alignment artifacts, (ii) Padding parameter in Shortstack, and (iii) Unifying the identified clusters from different serotypes to obtain the SRC. In the example in Supplementary Figure S2, one could see the interplay of all three reasons for the expansion of SRC localization. We decided to explore the effect of unification of clusters in introducing non-specific overlaps, as the other two reasons are not entirely with in our scope.

How many genes are introduced because of unification?

From the Supplementary Figure 2, we can observe that, we are introducing a lot of new distinct genes. There is a small effect on other annotation categories as well, but it is meagre.

How many clusters are introduced because of unification?

From the Supplementary Figure 2, we can see that because of the cluster merging we are introducing approximately 50% extra clusters in individual serotypes.

Boundary modification criteria

In order to correct for these non-specific partial overlap problems, we tried filtering SRCs with different overlap percentage to a gene.

Category-1: Should overlap at least 70% of a gene

This rather strict condition, removed all SRCs which overlap multiple genes and naturally, retained genes which overlap multiple SRCs. The table below shows the summary of SRCs, Genes and its overlaps with each other.

**Number of SRCs overlapping Multiple Genes**	
No. of SRCs	No. of overlapping genes
1687	1
**Number of Genes overlapping Multiple SRCs**	
No. of Genes	No. of overlapping SRCs
1243	1
120	2
38	3
12	4
6	5
2	6

Category-2: Should overlap at least 10% of a gene

This rather relaxed condition, retained most SRCs which overlap with multiple genes and a well as, retained genes which overlap multiple clusters. The table below shows the summary of SRCs, Genes and its overlaps with each other.

**Number of SRCs overlapping Multiple Genes**	
No. of SRCs	No. of overlapping genes
1643	1
189	2
11	3
1	5
**Number of Genes overlapping Multiple SRCs**	
No. of Genes	No. of overlapping SRCs
1645	1
123	2
36	3
11	4
3	5

Category-3:

As a trade-off, we tried altering the SRC boundaries for non-specific overlaps. i.e. In a gene-cluster overlap, if the gene is covered $> 80\%$ and the SRC is covered at least by 20%, then the SRC's boundary is limited to the gene's boundary. Doing so, limits the non-specific overlaps. But, this condition missed genes with multiple clusters being overlapped. So, we added another criteria. If the gene is covered $> 10\%$ and the cluster is covered more than 80%, those SRCs are retained with out any boundary changes. The table below shows the summary of SRCs, Genes and its overlaps with each other.

**Number of SRCs overlapping Multiple Genes**	
No. of SRCs	No. of overlapping genes
1335	1
32	2
1	3
1	4
**Number of Genes overlapping Multiple SRCs**	
No. of Genes	No. of overlapping SRCs
1283	1
87	2
15	3
2	4

As a results of these two conditions, we arrived at 1618 SRCs in total, which can be used for comparing with mRNA expression.

We overlapped these 1618 boundary modified SRCs against the 1445 GSRCs and removed the GSRCs which did not have any overlaps. We were left with 973 GSRCs after this non-specific overlap filtering.

2 Supplementary Figures

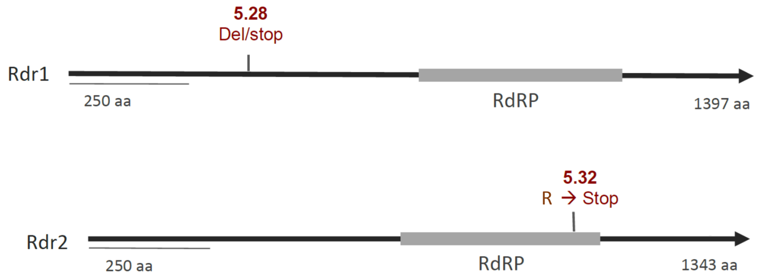

Figure S1: A scheme for the individual point mutations of the two RDR mutant lines. The black bar indicates the entire amino-acid sequence, in grey the RNA-dependent RNA-polymerase-domain (RdRP) The Rdr1 mutant shows a premature stop codon before the catalytic domain and the Rdr2 mutant inside the catalytic domain.

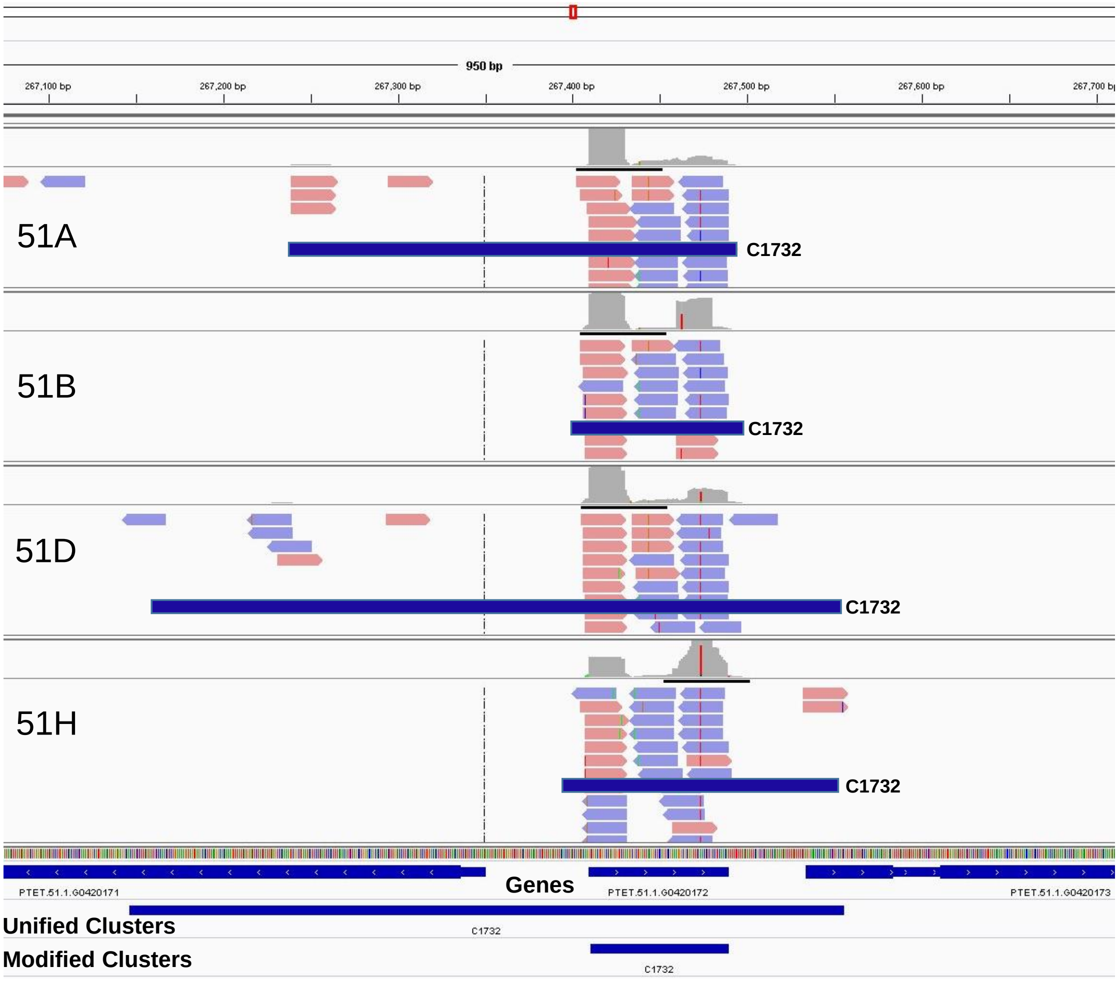

Figure S2: The localization of SRC C1732 in different serotypes is shown. In the bottom panel the SRC boundaries before and after modification along with the gene annotation is shown.

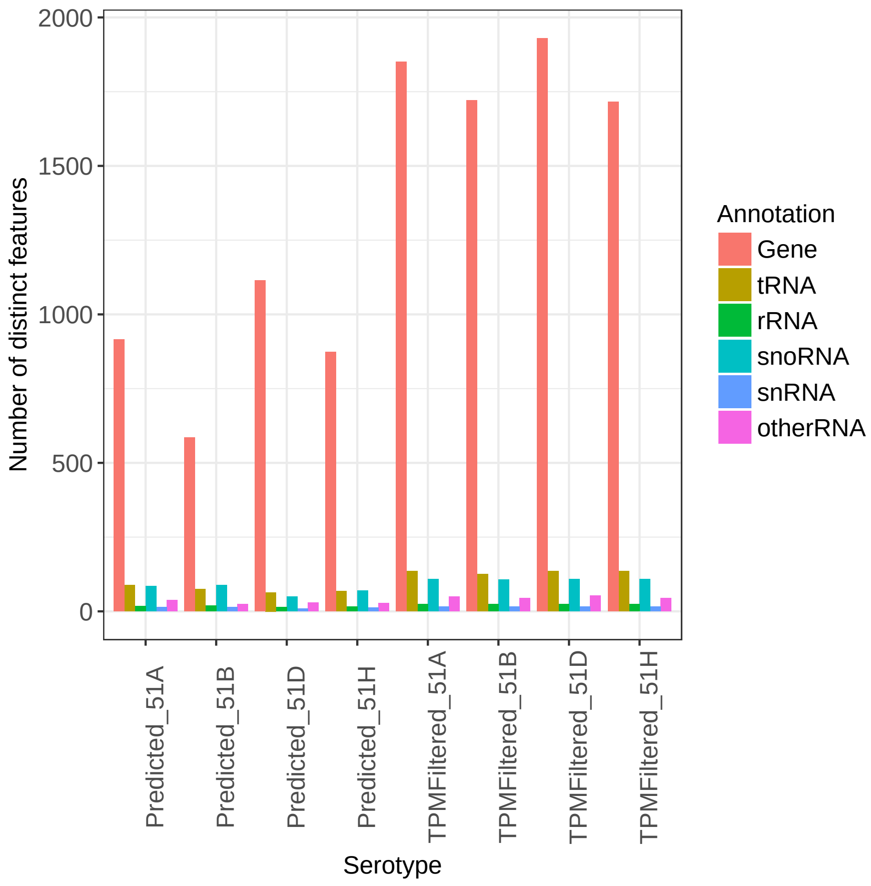

Figure S3: The effect of unification in the number of distinct features identified is shown. SRCs expressed in the wildtype serotype (51A, 51B, 51D, 51H; replicates were merged) samples were overlapped with annotated regions. Each annotated element is counted only once (distinct counting) and the number of elements of the different types (colors) is shown on the y-axis for all 4 serotypes. Predicted represents the distinct feature annotations while using the Shortstack's prediction results directly (i.e. Before unifying them to form SRCs). TPMFiltered represents what we call serotype specific SRC (i.e. SRC with a $TPM > 1$ in each serotype).

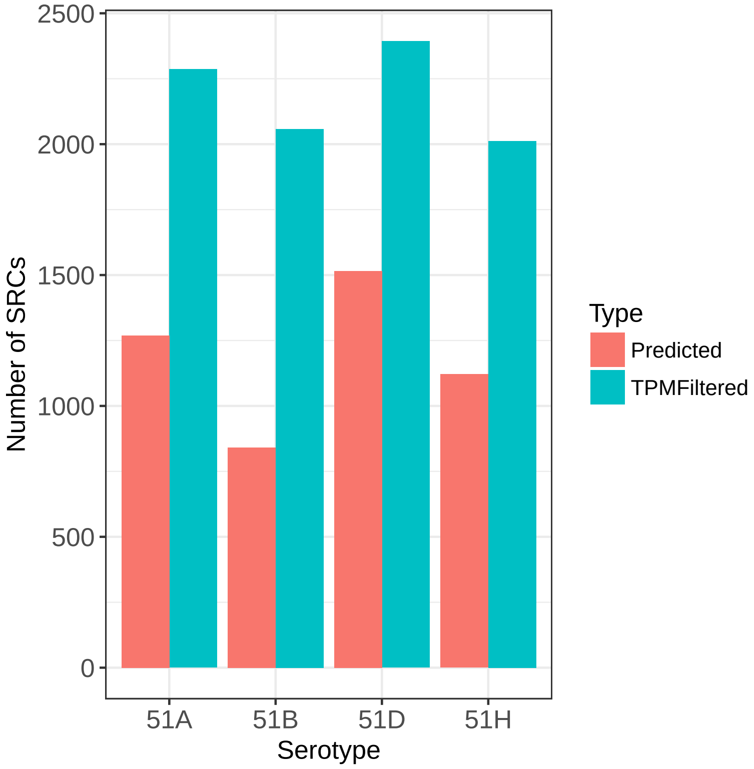

Figure S4: The number of SRCs identified in each wildtype serotype is shown. The type Predicted represents the distinct feature annotations while using the Shortstack's prediction results directly (i.e. Before unifying them to form SRCs). TPMFiltered represents what we call serotype specific SRC (i.e. SRC with a $TPM > 1$ in each serotype).

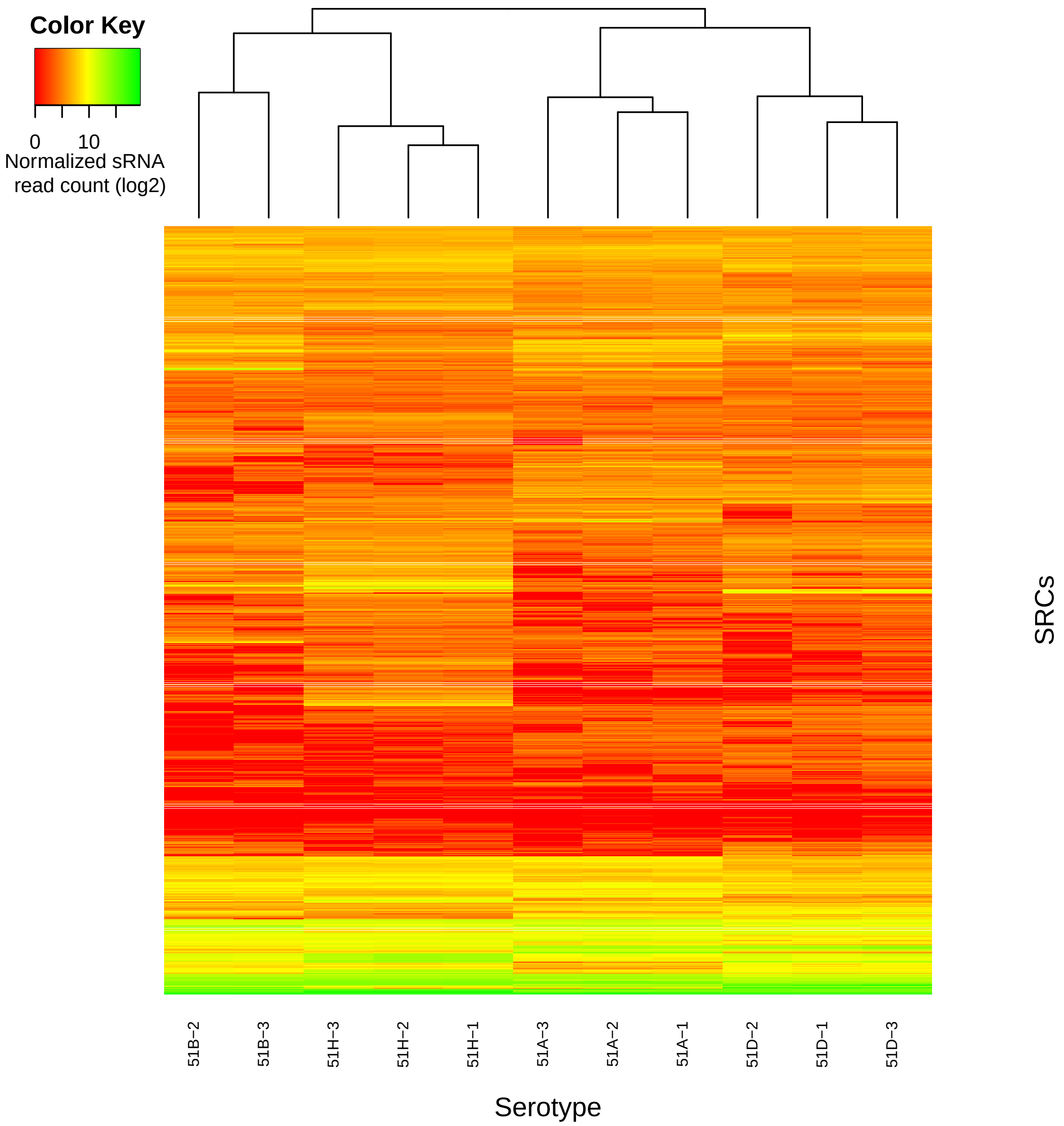

Figure S5: Heatmap of normalized sRNA expression after hierarchical clustering of all 2602 SRCs (rows) for all replicate experiments of the WT serotype samples (columns).

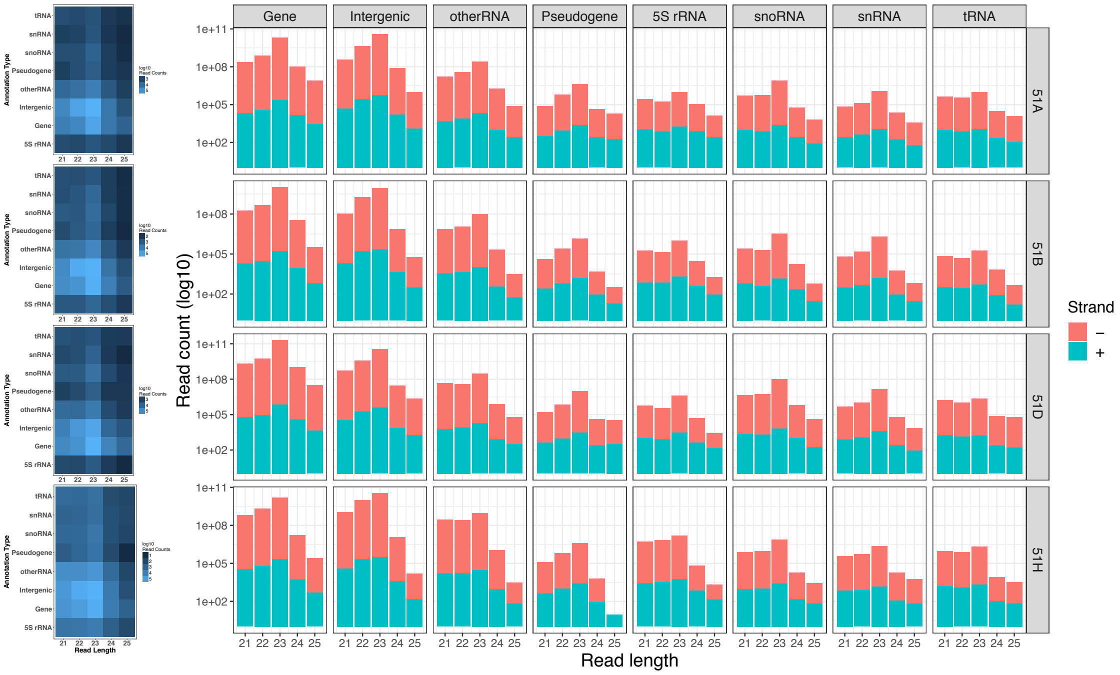

Figure S6: Supplementary figure for Figure 3. Left to right: A heatmap of sRNA accumulation (log10, color scale) in SRCs overlapping different genomic annotations and restricted to small RNA length (x-axis) for serotypes 51A, 51B, 51D and 51H is shown. Barplots showing the length distribution of sense (green) and antisense (red) sRNAs mapping to different genomic annotations in serotypes 51A, 51B, 51D and 51H is shown.

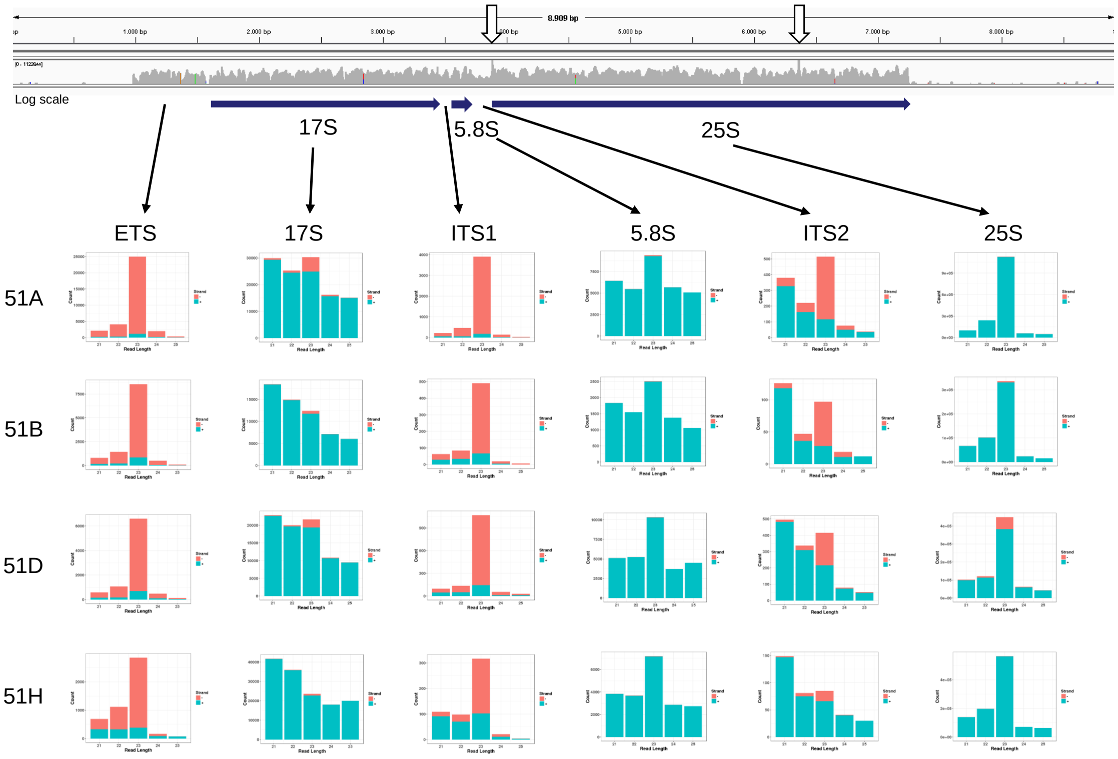

Figure S7: The read length distribution of small RNAs mapping to different regions of the ribosomal DNA is shown. Top: IGV screenshots of genomic localization of ribosomal DNA. Bottom: Each row corresponds to each wildtype serotype. Each column corresponds to ribosomal DNA regions in order: External Transcribed Spacer (ETS), 17S, Internal Transcribed Spacer 1(ITS1), 5.8S, Internal Transcribed Spacer 2 (ITS2), and 25S

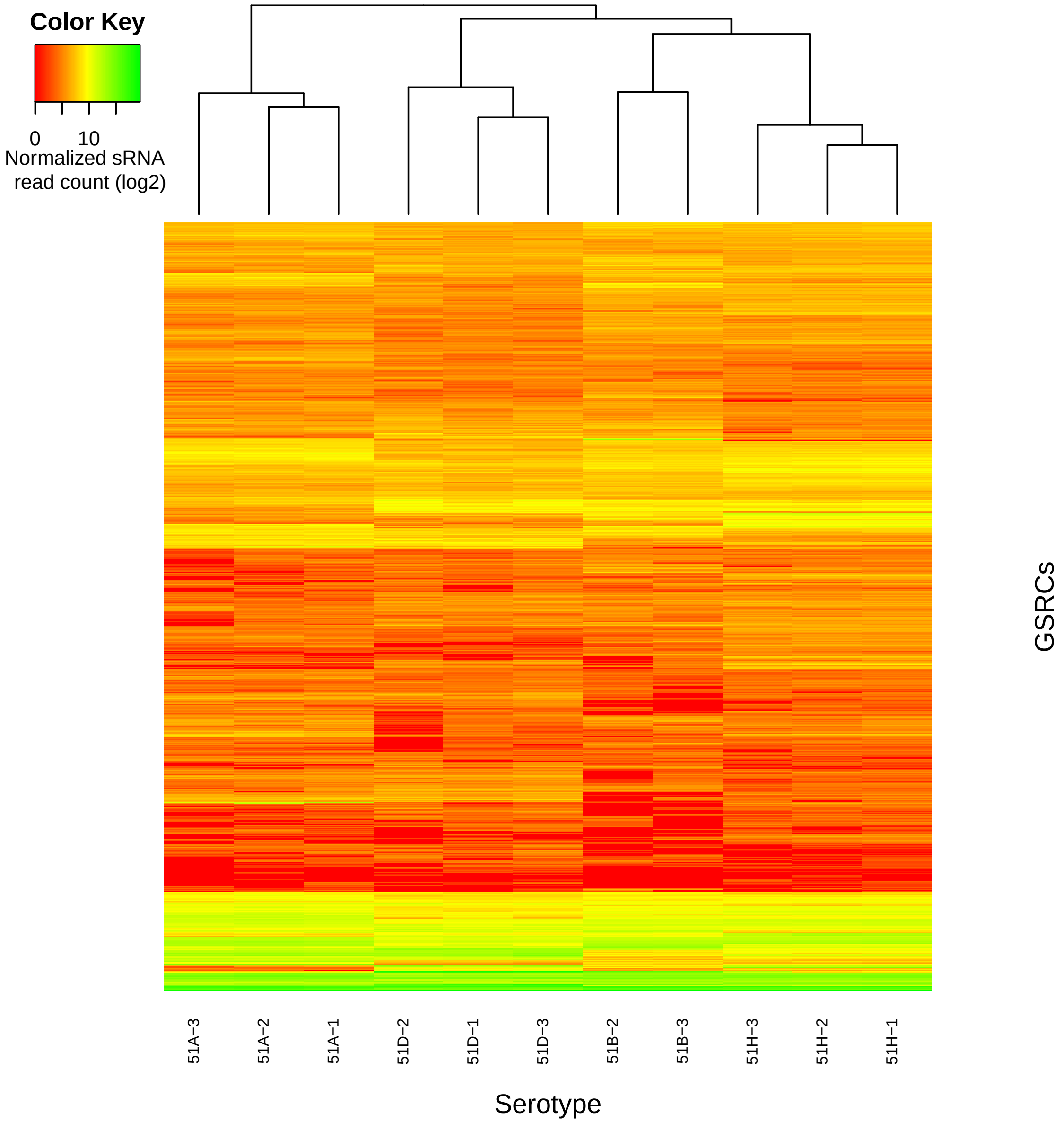

Figure S8: Heatmap of normalized sRNA expression after hierarchical clustering of all GSRCs (rows) for all replicate experiments of the WT serotype samples (columns).

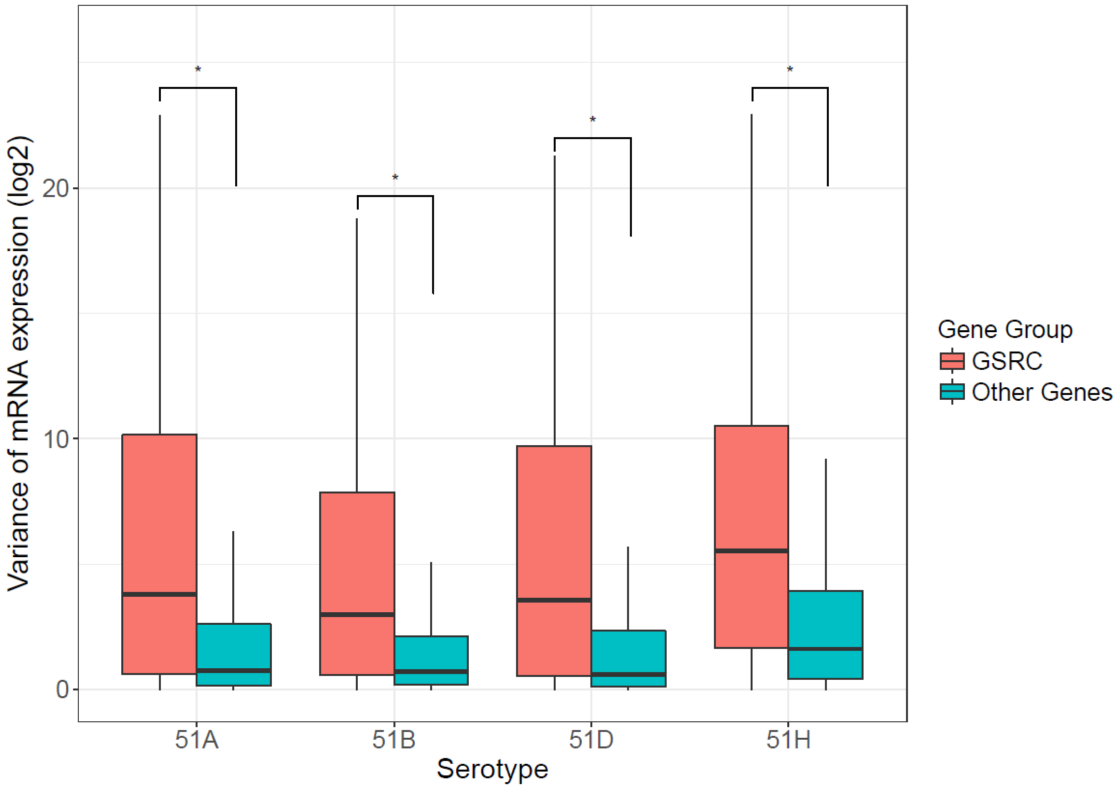

Figure S9: A) Boxplot of the variance of mRNA expression (y-axis, log2 TPM) in each wildtype serotype replicates of GSRCs, and the other expressed genes. We performed two-tailed wilcoxon test between GSRCs and others in respective serotypes, and found them to be statistically significant (p-value ¡ 0.05).

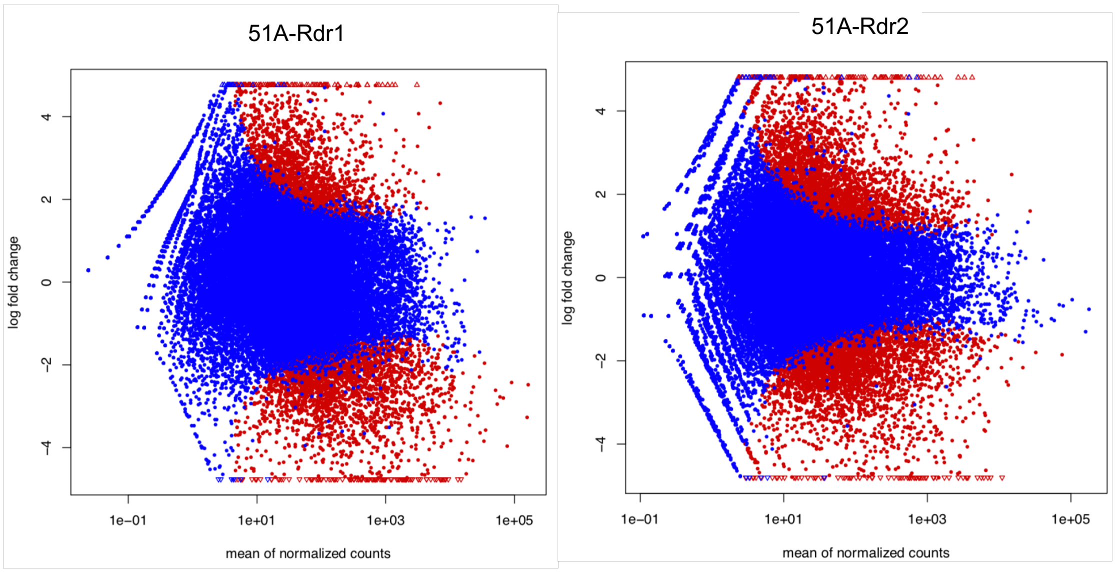

Figure S10: MA plots of 51A-Rdr1, and 51A-Rdr2 transcriptomes are shown here. The genes which are significantly differentially expressed in comparison with the WT 51A serotype, as identified by DESeq2, are shown in red.
